# Cognitive remediation therapy in low-and-middle-income countries (LAMICs) for people with psychosis: a systematic review of its efficacy on functional and cognitive outcomes

**DOI:** 10.1007/s00127-026-03136-x

**Published:** 2026-06-11

**Authors:** Yamini Vig, Yohannes Gebreegziabher, Charlotte Hanlon, Matteo Cella

**Affiliations:** 1 Institute of Psychiatry, Psychology and Neuroscience, King’s College London, London, UK; 2 Centre for Global Mental Health, London School of Hygiene and Tropical Medicine, London, UK; 3 Addictions and Related Research Group (ARG), Sangath, Porvorim, Goa India; 4 Department of Psychiatry, College of Health Sciences, Addis Ababa University, Addis Ababa, Ethiopia; 5 Department of Nursing, Asrat Woldeyes College of Health Sciences, Debre Berhan University, Debre Berhan, Ethiopia; 6 Division of Psychiatry, Centre for Clinical Brain Sciences, The University of Edinburgh, Edinburgh, Scotland, UK; 7 Department of Psychiatry, School of Medicine, College of Health Sciences, Addis Ababa University, Addis Ababa, Ethiopia; 8 South London and Maudsley NHS Foundation Trust, London, UK

**Keywords:** Cognitive remediation, Cognition, Psychosis, Severe mental illness, Low- and middle-income countries, Systematic review

## Abstract

**Purpose:**

Cognitive difficulties are commonly present in people with psychosis and are associated with poor functional outcomes. Evidence from high-income countries (HICs) indicates that cognitive remediation (CR) is an effective treatment for cognitive difficulties. Evidence from low and middle-income countries (LAMICs) is emerging. This study reviews the evidence for CR for people with psychosis in LAMICs.

**Methods:**

Seven databases (GIM, SCOPUS, WoS, APA PsycINFO, Global Health, Medline(R), and Embase) were searched. Two reviewers screened the studies retrieved independently against the eligibility criteria. Risk of bias was assessed using ROB 2.0 and ROBINS-I. The results on CR efficacy, alongside application and delivery, were synthesised narratively.

**Results:**

Fourteen studies from countries in Africa, Asia and South America were included. CR delivery modality and settings varied, including those delivered at home and by caregivers and health care workers. Thirteen studies showed that CR was beneficial for cognition, with small to large effect sizes. Nine studies showed that CR can improve functioning, with small to moderate effect sizes. The studies showed that CR was safe and well-tolerated. The effect of CR did not differ by setting, dosage, delivery agent or mode of delivery.

**Conclusions:**

CR could be a useful approach to reduce the burden of cognitive difficulties in people with psychosis in LAMIC. The adaptations to CR evaluated by the included studies could be used and further evaluated globally to provide novel, culturally acceptable and scalable implementation approaches for CR.

**Supplementary Information:**

The online version contains supplementary material available at https://doi.org/10.1007/s00127-026-03136-x.

## Introduction

People with psychosis present with complex and often pervasive difficulties that frequently impact long-term quality of life (QoL), as well as social and occupational functioning [1–4]. Access to good-quality mental health care is crucial for improving outcomes. In low-and-middle-income countries (LAMICs), there is limited access to timely, evidence-based mental health care, which means that people with psychotic disorders in LAMICs have a disproportionately higher likelihood of poor recovery prospects [5–8].

Since the earliest descriptions of psychotic disorders, cognitive difficulties have been recognised as a core feature and a major contributor to poor long-term outcomes [9–11]. Decades of research have demonstrated conclusively that people with psychotic disorders experience substantial cognitive difficulties across various cognitive domains, including attention, memory, processing speed, social cognition, and executive functioning [12–16]. Evidence has also linked these difficulties with poor social and occupational functioning [4, 12, 17]. Despite its clinical importance, the development of interventions targeting cognitive difficulties has lagged behind treatments for other symptom domains (e.g., psychotic symptoms), leaving a persistent gap in care [18, 19].

Considering the limited efficacy of currently available pharmacological approaches for cognitive impairment in psychosis, cognitive remediation (CR) has emerged as the intervention with the most robust evidence for targeting these deficits. CR is defined by the Cognitive Remediation Experts Workshop (CREW) as an intervention targeting cognition (attention, memory, executive functioning, social cognition, and metacognition) using the scientific principles of learning and with the goal of improving functional outcomes. Its effectiveness is enhanced when provided in a (formal or informal) context, providing support and opportunity for extending everyday functioning [20]. Extensive meta-analytic evidence suggests that CR can significantly reduce cognitive difficulties and improve functioning in people with psychosis, with small to moderate effect size [19, 21, 22], with treatment efficacy improving for CR approaches using four key ingredients: cognitive task practice, teaching strategy use, facilitating transfer and using trained therapists [20, 22, 23]. CR is also an acceptable intervention [24, 25], and there is evidence of its use in different settings and conditions [26–30].

Despite this evidence, the uptake of CR in routine mental health settings worldwide is still limited. This is due to various aspects, including poor training availability, resource constraints and assimilation with brain training programmes with poor and inconclusive evidence [31]. This poor uptake is also evident in LAMICs, perhaps for different reasons. CR is often delivered with the support of software requiring computers or tablets, often needing an internet connection, and LAMICs’ clinical services may find it difficult to access these resources [32–34]. Further, access to training material, therapy procedures and assessment tools for cognitive difficulties may be scarce due to limited training opportunities, translations, and validated assessment tools and therapy procedures [35, 36]. The limited use of culturally acceptable CR intervention strategies and tasks may further limit CR uptake. Lastly, the often-intense nature of CR delivery, with sessions offered up to 3–4 times per week, may be complex to achieve in clinical settings with limited human resources [35].

These barriers have not completely prevented the use and application of CR in LAMICs, with evidence emerging from the application of this approach in more and more countries [37–42]. These applications often adopted modifications to protocol in line with local needs, providing learning opportunities that can be used to extend the use and scalability of CR worldwide [37, 39]. However, there is a lack of systematic evidence about the feasibility and applicability of these approaches in LAMICs. Hence, this systematic review aims to summarise the current research on the efficacy of CR for people with psychotic disorders in LAMICs on cognitive and functional outcomes.

## Methods

### Review protocol

This review was conducted and reported based on the Preferred Reporting Items for Systematic Reviews and Meta-Analyses (PRISMA) guidelines [43, 44]. The protocol was registered on PROSPERO on 12th August 2024, ID: CRD42024578327. (https://www.crd.york.ac.uk/PROSPERO/view/CRD42024578327) (See Online Resource 1 for PRISMA Checklist).

### Search strategy

The initial search strategy was created on MEDLINE and adapted to the following databases: Embase, PsycINFO, Global Health (via OVID), Global Index Medicus, Scopus, and Web of Science. Searches were initially conducted in August 2024. Additionally, hand-searching was performed for studies and key reviews.

The search strategies were developed around the following concepts: cognitive remediation (Intervention), psychosis (Population), and low-and middle-income-countries (Setting). The Cochrane EPOC LAMIC (2023) filter was used for the LAMIC concept. (see Online Resource 2 for details on search strategies for all databases).

### Inclusion and exclusion criteria

Detailed inclusion and exclusion criteria are presented in Online Resource 3. For the population, the study focused on adults with a confirmed psychosis spectrum diagnosis resident in LAMIC (World Bank classification, 2024). Studies with participants experiencing prodromal psychosis, subclinical symptoms, or those at risk of experiencing psychosis were excluded. For intervention, we focused on CR approaches in line with the definition provided by the Cognitive Remediation Experts Workshop (CREW) 2023 [20], delivered both using computerised and non-computerised approaches and supported by a trained professional. Cognitive behaviour therapy and mindfulness-based interventions focused on improving cognition were excluded. Lab-based studies not intending to translate the intervention to healthcare settings were also excluded. Pre-post studies, and studies using treatment as usual, enhanced care, waitlist, passive or active control conditions were included. Studies comparing different CR modalities (e.g., one as active and one as control group) were excluded. For outcomes of studies, we included studies where primary outcomes were cognition and functioning, and secondary outcomes were adverse events, drop-out and quality of life (QoL). Experimental study designs with or without control groups were included; non-intervention, economic evaluation and psychometric studies, reviews, abstracts, posters, theses and protocols were excluded. There were no restrictions based on language and year of publication. The setting where care was delivered could be informal (e.g., places of worship, education institutes, etc.) or formal (primary, secondary or tertiary care).

### Study screening

Studies retrieved were deduplicated using Zotero and Rayyan [45, 46]. Two authors (YG and YV) independently screened all studies based on the eligibility criteria in Rayyan. The first stage involved title and abstract screening and then proceeded to the second stage—full-text screening.

Full texts were retrieved, and where necessary, authors were contacted to obtain access. As no language filters were applied, titles and abstracts, and if required—research papers in languages other than English were translated using Google Translate or DeepL [47, 48] and then screened against the eligibility criteria. Reasons for inclusion/exclusion were added to Rayyan through labels and/or notes. Conflicts identified post-full-text screening were resolved via discussions between YG and YV, and when conflicts persisted, senior authors (MC and CH) were contacted for resolution.

Attempts were made to translate studies via DEEPL and by approaching native speakers; however, due to the poor quality of translation, two studies were excluded.

**Fig. 1:**
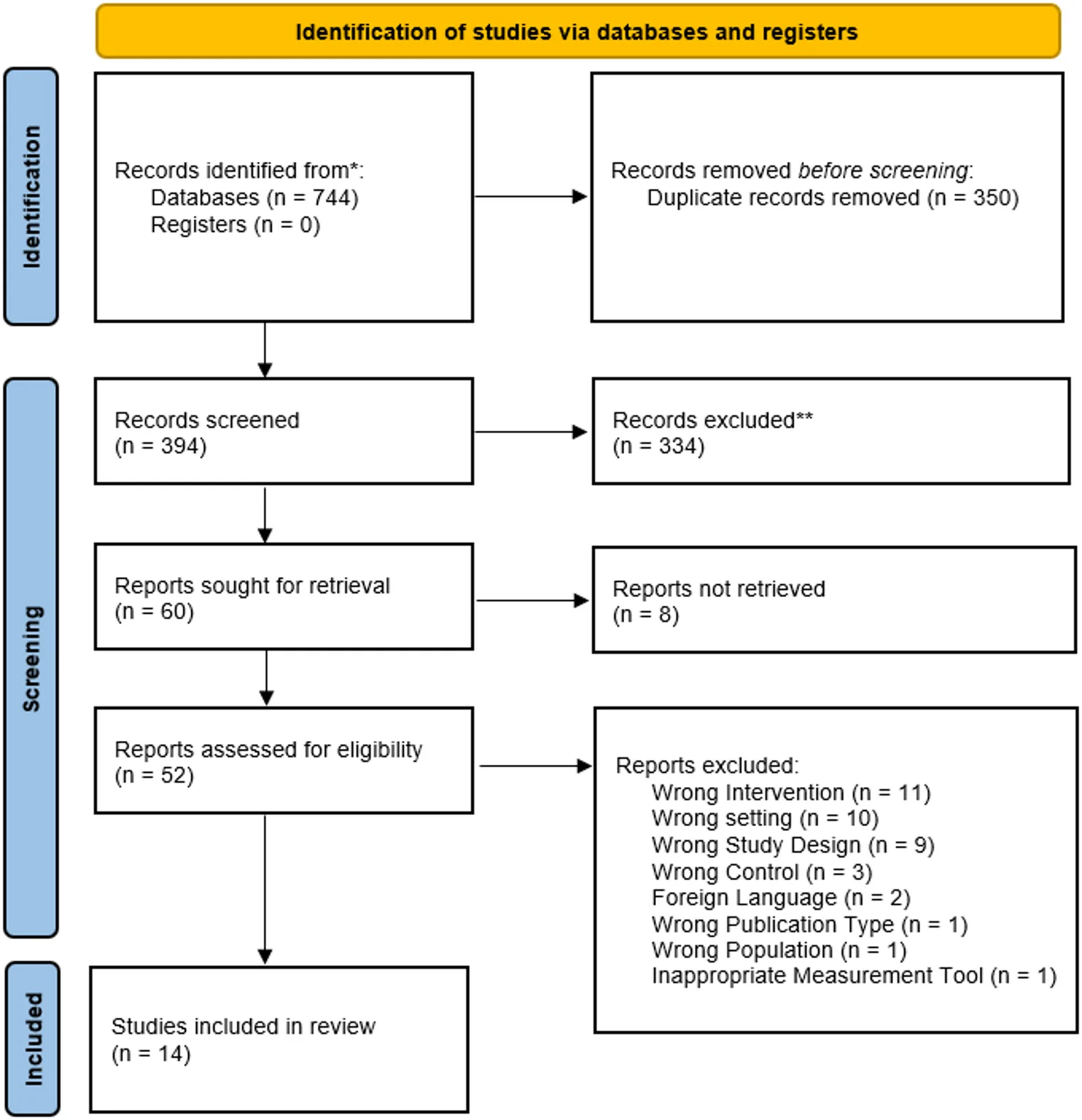
PRISMA Flow Diagram

The study selection process is summarised in the PRISMA flow diagram (Fig. 1) presented in the results section.

### Data extraction

Data were extracted via a customised excel data extraction template. Data extracted included bibliographic data, study characteristics, sample characteristics and intervention details. A comprehensive account of the data extracted can be found in Online Resource 4.

### Quality assessment

Quality assessment of the included studies was conducted using Cochrane’s Risk of Bias 2.0 (RoB 2.0) and the Risk of Bias In Non-randomised Studies of Interventions (ROBINS-I) [49, 50]. Cochrane’s guidance, handbooks, and crib sheet informed the quality assessment. Both tools required assessment based on the outcomes of each included study, which informed a risk of bias score for each study.

The RoB 2.0 involves assessing randomised studies on the following five domains: (1) randomisation process; (2) effect of assignment to interventions; (3) missing outcome data; (4) measurement of the outcome; and (5) selection of reported result. All domains are assigned a risk-of-bias judgement based on the signalling questions via an algorithm, i.e., “low” or “high” risk of bias or “some concerns”. The results from the domains inform an overall risk of bias judgment.

Similarly, ROBINS-I involves assessing non-randomised studies on the following domains: (1) confounding; (2) selection of participants into studies; (3) classification of interventions; (4) deviation from intended interventions; (5) missing data; (6) measurement of outcomes; and (7) selection of reported result. Based on the results, a risk of bias judgment is obtained for each domain, which in turn informs the overall risk of bias judgment for each study, i.e., “low”, “some concerns”, “moderate”, “high” or “serious”.

Figures showing the outcome of the quality assessment evaluation are available in Online Resource 5.

### Analysis and data synthesis

Information on study design, population (demographics, diagnoses, etc.), intervention characteristics (features of delivery, type of intervention, etc.), outcomes and quality assessment were summarised using narrative synthesis. This approach was selected as it was anticipated that the studies would be heterogenous with regards to sample characteristics, intervention components and characteristics, study context, setting and outcomes. This would make the use of meta-analytic procedures inappropriate.

## Results

### Searches

From an initial pool of 744 unique studies screened, we identified 52 eligible studies for full-text screening, of which 14 met the inclusion criteria. The PRISMA diagram is presented in Fig. 1.

### Study design

Of the 14 studies, 11 involved randomisation [37, 38, 40–42, 51–56], while three were non-randomised [39, 57, 58]. The three non-randomised studies offered assessment before-and-after therapy [57], a pilot non-randomised control trial [39] and a non-randomised control trial [58]. Of the 11 randomised studies, eight were randomised control trials (RCTs) [37, 38, 41, 42, 51, 53–55], including two multisite RCTs [41, 54]. The remaining three studies included pilot [40, 56] and feasibility RCTs [52].

### Study setting

Of the 14 studies included, 12 were based in Asia [37, 38, 41, 42, 51–58], and one each in Africa [39] and South America [40]. Seven studies were based in China [41, 51–55, 58], three in India [37, 38, 56] and two in Iran [42, 57]. Ten studies were based in upper middle-income countries [40–42, 51–55, 57, 58], three in lower middle-income countries [37, 38, 56], and one recruited both from lower middle-income and lower-income country contexts [39]. Most studies were conducted in tertiary care (teaching) hospitals or specialist psychiatric institutions [40–42, 51–58]. Two involved home-based interventions affiliated with tertiary hospitals [37, 38]. The remaining study was based in community-based psychiatric rehabilitation centres in West Africa [39] ( see Table 1 for study characteristics).

**Table 1:** Study Characteristics

Study	Study Design	Setting	Sample	Demographics					Attrition
				Age, years [ mean (sd)]	Gender (M/F)	Diagnoses (Clinical/Research)	Duration of Illness, years unless otherwise specified [mean (sd)]	Education, years, unless otherwise specified [mean (sd)]	Drop-outs
Hegde et al., 2012	RCT	Bangalore, India (Home-based)	45 (I: 22, C: 23)	I: 27.75 (5.25) C: 31(7.93)	I: 9/3 C: 10/1	Schizophrenia (ICD-10), clinical (confirmed by the consultant psychiatrist)	less than 2 years, NS	I: 14.33(3.84) C: 11.18(1.60)	14 /45 (I:9, C:5)
Lu et al., 2012	RCT	Third People’s Hospital of Lanzhou Municipality, Lanzhou, Gansu Province, China	I: 63, C: 63	I: 37(8), C: 38(9)	I: 40/23 C: 37/26	Schizophrenia (Chinese Classification and Diagnostic Criteria of Mental Disorders, third edition)	I: 23(9), C: 24(8)	I: 11(5), C: 10(4)	I: 3, C:1
Byrne et al., 2013	Feasibility study-randomised	Shanghai Mental Health Centre, China	I: 27, C: 24	I: 45.15(9.81), C: 46.04(8.68)	All male	Schizophrenia or schizoaffective (DSM-IV), clinical interview/review (treating psychiatrist)	I: 19.44(10.04) C: 24.92(8.82)	I: 11.93(3.77) C: 10.54(1.89)	I: 13, C: 7
Pontes et al., 2013	pilot RCT	Sao Paulo Medical School and the Integrated Mental Health Care Centre of Sao Paulo Hospital, Sao Paulo, Brazil	I: 9, C: 8	I: 37.1(8.1), C: 39.3(6.5)	I: 8/1, C: 6/2	Schizophrenia (DSM-IV-TR), psychiatrists	I: 15.1(7.8) C: 15.2(6.5)	I: 10(14.4), C: 10.7(12.7)	1, group not reported
Byrne et al., 2015	Non-randomised control trial	China, Shanghai; Shanghai Mental Health Centre	I: 20, C: 20	I: 38.3 (12.5) C: 37.0 (8.1)	40 males	Schizophrenia (DSM-IV), clinical interview & medical records	I:12.7 (5.6) C: 13.2 (8.6)	I: 11.6 (2.5) C: 11.7 (2.1)	Nil
Sharifi et al., 2016	Before-After Study (intervention)	Roozbeh Hospital (Tehran City, Iran)	20	35.5(8.3)	16/04	Schizophrenia (DSM IV), clinical (psychiatrist, confirmed via DSM-IV SCID Persian version)	135.3(93.4) months	1 (8th grade), 1 (10th grade), 11 (high school diploma), and 7 (college/university degree)	Nil
Tan et al., 2016	RCT	Beijing Huilongguan Hospital, Beijing, China	I: 44, C: 46	I: 46.77(7.18) C: 46.09(5.52)	I:27/17, C:27/19	Schizophrenia (DSM-IV), clinical (psychiatrists)	I: 23.95(8.18) C: 21.51(6.50)	I: 9.70(1.85), C: 10.13(2.42)	I: 8, C:6
Tan et al., 2020	multisite RCT	Beijing Huilongguan Hospital, Beijing Anding Hospital and Peking University Sixth Hospital (China)	I: 196, C: 115	I: 45.60(8.68) C: 44.24(8.31)	I: 122/74, C: 69/46	Schizophrenia (DSM-IV), clinical (psychiatrists)	I: 21.16(9.77), C: 19.18(9.44)	I: 11.74(2.82) C: 11.86(2.72)	I: 24, C: 19
Zhu at al., 2020	Multi-centre RCT	China, Beijing	I: 78, C:79	I: 43.74(9.24), C: 43.65(8.64)	I: 42/36, C: 43/36	Schizophrenia (DSM-IV), clinical	I: 19.51(9.45), C:17.71(9.57)	I: 11.46(2.37), C: 11.77(2.58)	I: 5, C: 13
Hatami et al., 2021	RCT	Roozbeh Hospital (Tehran City, Iran)	I: 31, C: 31	I: 35.9(8.5) C: 38.9(9.5)	Not Mentioned	Schizophrenia or schizoaffective disorder (DSM-IV-TR), clinical-Persian version of structured clinical interview for DSM-IV (SCID-I)	I: 150.7(87.5) and C: 141.6(100.5) months	Not Mentioned	I: 3, C:3
Zhu et al., 2021	RCT	Beijing Huilongguan Hospital, China	CCRT: 144, CRT: 72, & Active Control: 54	CCRT: 46.60(8.94), CRT: 47.56(8.23) & Active Control: 46.11(8.21)	CCRT: 91/53, CRT: 48/24, & Active Control: 33/21	Schizophrenia (DSM-IV), two psychiatrists	CCRT: 22.78(9.87), CRT: 24.14(9.96) & Active Control: 22.18(10.22)	CCRT: 11.24(2.71), CRT: 11.76(2.30) & Active Control: 11.41(2.55)	CCRT: 18, CRT: 8, & Active Control: 4
Moun et al., 2022	RCT	Tertiary Care Hospital, North India (Homebased)	I: 36, C: 38	I: 29.79(6.5), C: 29.48(5.7)	Not reported	Schizophrenia (ICD-10 DCR), clinical (consultant psychiatrist)	Not reported	I: 12.79(3.39), C: 13.21(2.90)	I: 8, C: 9
Deste et al., 2023	Pilot controlled study; non-randomised	Togo and Benin (specialised secondary care setting)	36 (I:18, C:18)	Age: 31.08 (8.47) I: 29.78(9.56), C: 32.39(7.27)	27/09	Schizophrenia (DSM-5), clinical (performed by medical doctor, assisted by psychiatrist when required)	mean 10.6 ± 15.8, I: 8.13(11.21) C:14.70(21.36)	10.3 ± 2.3, I: 11.50(2.26) C: 9.17(1.82)	Nil
Singh et al., 2023	Preliminary RCT	Department of Psychiatry, Chandigarh, Panjab, India	I: 15, C: 14	I: 37.1(7.7) C: 39.7(5.9)	I (8/7) C (9/5)	Chronic Schizophrenia (ICD-10)	I: 10.5(8) C: 11.1(8)	I: 16.8(3.04) C: 17.9(2.16)	Nil

SD, standard deviation; SE, standard error; I, intervention group; C, Control Group; DSM, Diagnostic and Statistical Manual of Mental Disorders; ICD, International Classification of Diseases; RCT, Randomised Control Trials. Where studies reported SE, values were converted into SD using SD = SE x$\:\sqrt{n}$

### Study sample

The sample size of the included studies ranged from 20 to 311. Two studies did not report the gender of the sample included in the studies [38, 42], and of the studies that reported the gender, all had a sample with more males than females. Five studies reported the mean age of the sample to be 40 years and above [41, 52–55], while the rest had a sample of people within a mean age ranging between 27 and 40 years [37–40, 42, 51, 56–58]. One study did not report the level of education [42], of the thirteen that reported education in years this ranged from 9.7(SD1.85) to 16.8(SD3.04) for the intervention group, and 9.17(SD1.82) to 17.9(SD2.16) for the control group [37–41, 51–58]. Twelve studies recruited exclusively people with a schizophrenia diagnosis [37–41, 51, 53–58]. Two studies mentioned both people with schizophrenia and schizoaffective disorder in the eligibility criteria [42, 52]. Ten studies used a variation of the Diagnostic and Statistical Manual (DSM) to confirm the diagnoses, i.e., DSM IV, DSM IV-TR and DSM 5 [39–42, 52–55, 57, 58], while three used the International Classification of Diseases (ICD-10) [37, 38, 56]. One study used a contextualised tool to diagnose schizophrenia, i.e., the Chinese Classification and Diagnostic Criteria of Mental Disorders, third edition [51]. Thirteen studies reported the duration of illness; ten studies reported it through mean and standard deviation in years [39–41, 51–56, 58], with the mean age ranging from 10 to 24 years, two reported it in months [42, 57], and one reported it to be less than two years for all participants [37].

### Attrition

Four studies reported no dropouts [39, 56–58]. Nine studies reported cumulative dropouts as well as dropouts for CR and the chosen control condition, while one study did not report the group participants dropped out from [37, 38, 41, 42, 51–55]. The attrition rate ranged from 3.2% to 39.2%, with studies reporting participants’ discharge, deterioration of symptoms, relocation, unavailability of caregivers or change in employment status as contributing factors.

### Care settings

Twelve studies delivered interventions in specialised mental health care settings, of which six recruited inpatients of the respective facilities [39, 41, 51–53, 55], four studies recruited outpatients [40, 54, 56, 58] and two studies did not mention the nature of the service type [42, 57]. The remaining two studies were conducted in participants’ homes with intermittent supervised sessions in the local hospital [37, 38].

### CR core features

Tables 2 and 3 offer a detailed overview of intervention characteristics and core therapy features included in this review.

**Table 2:** Intervention Characteristics

Study	Intervention	Comparator	Mode/ Platform/ Delivery Agent	Duration, Intensity and Frequency	Cognitive domains & Measurement tools	Assessors (Reliability and Validity)	Functional Outcomes and Tools	Assessors (Reliability and Validity)
Hegde et al., 2012	Home-based cognitive retraining + Psychoeducation; 2 Sets of cognitive exercises covering multiple domains (subjective questions)	Drug treatment + Psychoeducatrion	Manualised; Homebased (2 check-in meetings post-initiation). Monitoring from caregiver, supervision from researchers	15 tasks over two months, set 1 (weeks 1–4), Set 2 (weeks 5–8), and three Psychoeducation sessions (45–60 min) at preintervention, 1-month follow-up and post-intervention	Attention (DVT, Colour trails test & triads test), Motor speed (finger tapping test), Mental Speed (DSST), Verbal Comprehension (token test), EF (Animal names test, verbal N-back task, Stroop test, ToL, WCST, Verbal Learning & memory (RAVLT) and Visuo-constructive ability and visual memory (Rey Osterrieth-CFT).	Researcher (NS)	Disability in overall functioning (WHO Disability Assessment Schedule-II, WHODAS-II)	Researcher [NS]
Lu et al., 2012	Chinese CRT targets cognitive flexibility, WM & planning	TAU (Medicines+ recreational therapy-dance, music counselling/ mental health education-same frequency, intensity & duration as Intervention)	Manualised; Hospital; 4 trained therapists	45-minute sessions, five days/week for three months	Cognition (WCST)	2 trained psychologists [ICC(WCST) = 0.80]	Social Functioning (Scale of Social Skills of Chronic Schizophrenia Inpatients)	2 trained psychologists [internal consistency (alpha = 0.89), test-retest (*r* = 0.97), IRR (*r* = 0.98) and ICC = 0.87]
Byrne et al., 2013	Cross-cultural CDT Program. Targets attention, visual WM (spatial WM task & pair matching) & verbal WM (arithmetic, digit & word span). in Chinese & English-exercises in arithmetic, number lists, pair matching, spatial WM & word lists.	TAU (medicines)	Computerised; Mental Health Centre. RA assisted by a Psychiatric nurse	1 orientation session + at least 12 sessions (30–60 min). 2–3 sessions/week over. Mean sessions completed = 12.78.	Verbal memory & learning (Chinese version of letter-number sequencing task), verbal memory (Form B, HKLT, Chinese language)	RA with assistance from a psychiatric nurse (Validated for Chinese sample)	Global Functioning (Severity of Illness item, CGI) and Social Functioning (Chinese version of PSF)	RA assisted by psychiatric nurse [PSP (internal consistency-Cronbach’s alpha = 0.84; test-retest reliability-ICC = 0.95; IRR-kappa value = 0.82)]
Pontes et al., 2013	Cognitive Attention & Memory Training. Progressive difficulty levels with first 10 focused on Attention & 10 on memory. CAMT targets metacognition, contextualisation, verbalisation & positive reinforcement.	Active Control (Reading training in same frequency, duration & intensity)	Manualised (Pen & Paper); Hospital. Researcher	40–60-minute group sessions per week, over 5 months, total sessions = 20 (1 session/week)	TMT, Stroop Colour Naming Test, Modified WCST, & Logical Memory & Visual Reproduction (WMS-III)	Specialist neuropsychologists [NS]	NA	NA [NA]
Byrne et al., 2015	Cross-cultural CDT Program. Targets attention, visual WM (spatial WM task & pair matching) & verbal WM (arithmetic, digit & word span). in Chinese & English-exercises in arithmetic, number lists, pair matching, spatial WM & word lists+ computerised FAR Module. 24 photographs (Chinese ethnicity), 6 emotions, participant selects emotion which describes facial affect.	TAU (medicines)	Computerised; Mental Health Centre. Group sessions monitored by 2 psychiatric nurses	45–60-minute sessions, post-assessment, mean sessions attended ~ 12.85 (CDT) & FAR (12.60) over 6-weeks	Verbal memory & learning (Chinese version of letter-number sequencing task), verbal memory (Form B, HKLT, Chinese language) + Number of correct matches of emotions to photographs (scored 0–24).	psychiatric nurses or researcher (Validated for Chinese sample)	Global Functioning (Severity of Illness item, CGI) and Social Functioning (Chinese version of PSF)	psychiatric nurses or researcher [PSP (internal consistency-Cronbach’s alpha = 0.84; test-retest reliability-ICC = 0.95; IRR-kappa value = 0.82)]
Sharifi et al., 2016	CCRT (Cogpack-attention, memory, distributive, selective & sustained attention, short-term memory, long-term memory & working memory, planning & problem-solving)	NA	Computerised; Hospital. Supervised by master’s level supervisor	10 one-hour sessions, 2–3 times/week (in-patients) & 2 times per week (out-patients).	CANTAB, Attention (SST and Choice Reaction Time), Memory (PRM & PAL test) & EF (Stockings Exchange of Cambridge test and IED task)	Master’s level assessor (NS)	Global Functioning (GAF)	Master’s level assessor [NS]
Tan et al., 2016	Group CRT via the Chinese version, CRT (Frontal/EF Program revised); 3 modules, targets cognitive flexibility, WM capacity & planning	Musical & Dance Therapy, same frequency, duration & intensity as intervention group	Manualised (Pen and Paper); Hospital. 4 trained therapists conducted sessions with 3–4 patients/group.	40 hourly sessions, mean ~ 4 per week.	Assessment via: Stroop Neuropsychology Screening Test, Category Fluency Test (CFT), Verbal fluency Test (VFT), TMT-A, Logical Memory Test, Benton Visual Retention Test (BVRT) & Digit Span (WAIS-R)	2 clinical psychologists (NS)	Behaviour and Social Functioning [Nurse’s Observational Scale for Inpatient Evaluation (NOSIE-30)]	4 senior nurses [NS]
Tan et al., 2020	Group CCRT System	Learning to dance/easy musical instrument+ motivation & positive feedback from a therapist; same frequency, intensity & duration as CCRT	Computerised; Hospital. Trained therapist, 1 therapist per 4 participants	45-minute sessions 4–5 times per week for 12 weeks, totalling 50 h	PS (Category Fluency Test, TMT-A & Symbol Coding Test), Attention (Continuous Performance Test-Identical pair), WM [Spatial Span Test (Wechsler Memory Scale-3rd edition), Digit Sequencing test], Verbal Learning (Hopkins Learning Test-Revised, Chinese version), Visual Learning (Brief Visuospatial memory Test-revised), Reasoning & Problem Solving [mazes test, NAB-MAZES)] & Social Cognition (MSCEIT, Chinese version). Secondary outcomes: verbal WM (WAIS-III: digit span test) & EF (WCST).	4 trained clinical psychologists [IRR (k = 0.80)]	Functional Capacity (Chinese version of the UCSD Performance-based Skills Assessment) & behavioural and functional capacity (Chinese version of NOSIE-30)	Four senior nurses [IRR = 0.85]
Zhu et al., 2020	CCRT via software. 30 activities/session which target cognitive flexibility, WM, planning & social functioning. Delivered in groups, i.e., 4 participants supervised per therapist.	TAU (anti-psychotics)	Computerised; Hospital. Trained therapist, 1 therapist per 4 participants	45-minute sessions 4–5 times per week for 12 weeks, totalling 50 h	PS (Category Fluency Test, TMT-A & Symbol Coding Test), Attention (Continuous Performance Test-Identical pair), WM [Spatial Span Test (Wechsler Memory Scale-3rd edition), Digit Sequencing test], Verbal Learning (Hopkins Learning Test-Revised, Chinese version), Visual Learning (Brief Visuospatial memory Test-revised), Reasoning & Problem Solving [Mazes test (NAB-MAZES)] & Social Cognition (MSCEIT, Chinese version). Secondary outcomes: verbal WM (WAIS-III: digit span test) & EF (WCST).	4 trained clinical psychologists [intra-rater reliability (k = 0.80)] [MCCB (test-retest reliability = 0.88, diagnostic validity = 0.85), IRR (kappa = 0.90)]	Social and Occupational Functioning (Personal & Social Performance) scale and Day-to-day functioning (Chinese version of the USCD Performance-based Skills Assessment)	4 trained clinical psychologists [PSP (test-retest reliability = 0.95, internal consistency = 0.84) and UPSA (test-retest reliability = 0.75 and diagnostic consistency = 0.72)]
Hatami et al., 2021	Medicines + CCRT. CRT via modified Cogpack (21/64 tasks suited to an Iranian population). Targets attention, visual memory and EF.	TAU (Pharmacotherapy + Psychoeducation)	Computerised; Psychiatric centre. Psychologists/ social workers/ occupational therapists supervised by psychiatrist	60–75 min sessions, 2–3 times/week, 10 sessions	Some tests of CANTAB 3.2: Attention (SST & CRT), Visual memory (PRM & PAL) and EF (Stockings of Cambridge, Stop-Signal Task and IED)	Trained psychologist + psychiatry resident (NS)	Functioning (GAF & CGI) & QoL (Persian version of the WHO QoL-Brief version)	Trained psychologist + psychiatry resident [NS]
Zhu et al., 2021	Arm 1 (CCRTS): CCRT System via software, targeting cognitive flexibility, WM and planning + 3 social cognition exercises. 6–25 tasks/exercise || Arm 2 (CRT): Chinese version of a Pen & Paper CRT targeting cognitive flexibility, WM & planning.	Active Control (Learning to play a simple instrument/dance)	Arm 1: Computerised; Arm 2: Manualised. Hospital Arm 1: 1 therapist per 4 patients; Arm 2: 4 trained therapist, each supervising 3–4 participants	45-minute sessions 4–5 times per week for 12 weeks, totalling 50 h	PS (Category Fluency Test, TMT-A & Symbol Coding Test), Attention (Continuous Performance Test-Identical pair), WM [Spatial Span Test (Wechsler Memory Scale-3rd edition), Digit Sequencing test], Verbal Learning (Hopkins Learning Test-Revised, Chinese version), Visual Learning (Brief Visuospatial memory Test-revised), Reasoning & Problem Solving [Mazes test (NAB-MAZES)] & Social Cognition (MSCEIT, Chinese version). Secondary outcomes: verbal WM (WAIS-III: digit span test) & EF (WCST).	4 trained clinical psychologists [intra-rater reliability (k = 0.80)] [MCCB (test-retest reliability = 0.88, diagnostic validity = 0.85), IRR (kappa = 0.90)] [Cognition (Internal consistency kappa = 0.90)]	Functional Capacity (Chinese version of the UCSD Performance-based Skills Assessment) & behavioural & functional capacity (Chinese version of NOSIE-30)	4 senior nurses [kappa = 0.85]
Moun et al. 2022	Home-based CT + TAU. (Attention, mental speed, verbal fluency, WM & EF (response inhibition, planning, set shifting ability & abstraction). 4 sets delivered, 1st post-baseline assessment & subsequently at 2-week intervals.	TAU (medicines + psychoeducation)	Manualised; Homebased (therapists demonstrated new activities in the hospital every 2 weeks). Monitoring from caregiver, supervised by researchers	6 days/week for 8 weeks. Therapist supervised sessions every two weeks (60–90 min). Set manuals contained tasks designated per day.	Brief Assessment of Cognition in Schizophrenia (BACS): Verbal Memory, Digit Sequencing, Token Motor, Verbal Fluency, Symbol Coding, ToL.	Researcher (NS)	Disability (WHODAS 2.0) & QoL (WHOQOL-BREF)	Researcher [NS]
Deste et al., 2023	CRT in French (Cognitive Flexibility, Memory and Planning Modules)	TAU (Medicines+ rehabilitation activities) + g	Manualised (Pen and Paper). Psychiatric Health Centres. Mental Healthcare Workers	40 one-hour sessions thrice a week over ~ 14 weeks	PS (TMT-A), WM (TMT B, WAIS-R Digit Span Backwards), EF & CF (TMT B-A), PS (WAIS-R Digit Symbol Coding) and Verbal Learning and Memory (RAVLT)	Trained psychologists not involved in CRT delivery. (IRR = 0.8)	Real-world functioning (GAF)	Trained psychologists not involved in CRT delivery [IRR = 0.8]
Singh et al., 2023	CCT (‘Cogbrain”) targets attention, PS, visual memory, WM, EF & verbal memory. (In Hindi, Punjabi & English)	TAU (Medicines+ psychosocial support-community social workers, occupational rehab-arts & crafts, group activities, discussions & peer-support)	Computerised (7-inch tablets). Hospital, department of psychiatry. Trained therapists	1-hour sessions, 4 days/week for 3 months	NIMHANS Neuropsychological Battery. Attention (trail-making, triads & DVT), PS (DSST), Verbal fluency (controlled oral word association test), category fluency (animal naming test), set shifting (WCST), response inhibition test (ToL & Stroop test), verbal & visual WM (N-Back test), Verbal learning and memory (Auditory verbal learning test, passages test & Rey CFT)	Trained clinical psychology assistants (NIMHANS neuropsychological battery is culturally adapted to assess an Indian population)	Socio-functional outcomes (Social Occupational Functioning Scale) & Activities of Daily Living (Lawton instrumental activities of daily living scale)	Trained clinical psychology assistants [NS]

TAU, treatment as usual; SE, standard errors; EF, executive function; WCST, Wisconsin Card Sorting Task; AVLT, Auditor Verbal Learning Task; CFT, Complex Figure Test; WHODAS, WHO Disability Assessment Schedule; SD, standard deviation; SSSI, Scale of Social Skills of Chronic Schizophrenia Patients; LNS, Letter Number Sequencing; HKLT, Hong Kong List Learning Test; WM, working memory; TMT-A, Trail-Making-Test-A; TMT-B, Trail Making Test-B; TMT B-A, Trail Making Test B-A; MWCST, modified Wisconsin card sorting test; SOC, Stocking of Cambridge; IED, intra-extra dimensional task; SST, stop signal task; BVRT, Benton Visual Retention Test; NOSIE, Nurse’s Observation Scale for Inpatient Evaluation; MCCB, MATRICS Consensus Cognitive battery; HVLT-R, Hopkins Verbal Learning Test-revised; NAB-Mazes, Neuropsychological Assessment Battery-Mazes; MSCEIT, Mayer-Salovery-Caruso Emotional Intelligence Test; PS, Processing Speed; CRT, Cognitive Remediation Therapy; CCRT, Computerises Cognitive Remediation Therapy; PAL, Paired Associate Learning; GAF, Global Assessment of Functioning; WHOQOL-BREF, WHO Quality of Life-Brief version; WAIS-R, Weschler’s Intelligence Scale-revised; NS, not-specified; NA, not applicable; RAVLT, Rey Auditory Verbal Learning Test; CANTAB, Cambridge Neuropsychological Test Automated Battery; PRM; Pattern Recognition Memory; ToL, Tower of London; CDT, Computer Drill Training; PSP, Personal and Social Performance Scale; CGI, Clinical Global Impressions; FAR, Facial Affect Recognition; min, minutes; QoL, Quality of Life; UCSD, University of Southern California, San Diego Performance Based Skills Assessment; NIMHANS, National Institute of Mental Health and Neurosciences; VT, Digit Vigilance Test; DSST, Digit Symbol Substitution Test; RA, Research Assistant; ICC, Intraclass Correlation Coefficient; IRR, Inter-Rater Reliability

**Table 3:** Core therapy features

Studies	Therapy Features				Techniques			Culturally Contextualised Tasks
	Cognitive Exercises	Cognitive Strategies	Active Therapist	Transfer to the real world	Massed Practice	Scaffolding	Errorless Learning	
Hegde et al., 2012	X				X	X		X
Lu et al., 2012	X	X	X	X	X	X	X	
Byrne et al., 2013	X		X		X	NS	NS	
Pontes et al., 2013	X	X	X	X	X	X	NS	
Byrne et al., 2015	X		X		X	NS	NS	
Sharifi et al., 2016	X		X		X	X	NS	
Tan et al., 2016	X	X	X	X	X	X	X	
Tan et al., 2020	X	X	X	X	X	X	X	
Zhu et al., 2020	X	X	X	X	X	X	X	
Hatami et al., 2021	X		X		X	X	NS	
Zhu et al., 2021	X	X	X	X	X	X	NS	
Moun et al., 2022	X				X	X		X
Deste et al., 2023	X	X	X		X	X	X	
Singh et al., 2023	X		X		X	X	X	

NS, Not Specified

Massed practice emerged as a common ingredient across all included studies. Massed practice refers to the repeated and intensive rehearsal of cognitive exercises across multiple sessions [23]. This is influenced by session frequency, duration, and intensity, which varied significantly in the studies considered. Seven studies explicitly mentioned teaching the use of cognitive strategies to the participants [39–41, 51, 53–55]. Except for two home-based studies [37, 38], all studies were delivered under the supervision of an active therapist [39–42, 51–58]. Notably, only the two home-based studies included culturally contextualised tasks within the remediation package. Twelve studies explicitly stated that their interventions used scaffolding principles [37–42, 51, 53–57], the remaining two studies did not specify [52, 58]. Six studies reported using errorless learning task practice [39, 41, 51, 53, 54, 56], while six did not specify [40, 42, 52, 55, 57, 58]. No study clearly described efforts to transfer CR learning and gains to real-world settings.

### Mode of delivery

Seven studies employed computer software to deliver the interventions [41, 42, 52, 54, 56–58], while six involved manualised/pen-paper-based delivery [37–40, 51, 53]. One study had two intervention groups, receiving computerised and manualised training, respectively [55]. Among the studies involving computerised methods, one study utilised tablets to deliver a custom-made application ‘Cogbrain’ based on CR principles [56]. Software utilised by studies included ‘Cogpack’ [42, 57], a cultural adapted ‘Computerised Drill Training’ [52, 58], ‘Computerised Cognitive Remediation Therapy System’ [41, 54, 55] and ‘Cognitive Attention and Memory Training’ [40]. Seven studies reported intervention delivery in groups [40, 41, 52–55, 58]. The therapy language included English, French, Chinese, Hindi, Punjabi, Kannada, and Persian.

### Delivery agent

Six studies employed trained therapists to deliver interventions [41, 51, 53–56]. Four studies used allied health professionals (AHPs), i.e., social workers, psychiatric nurses, mental health workers, and occupational therapists [39, 42, 57, 58]. Two involved research staff administering the interventions [40, 52], while caregivers of participants delivered the interventions in the remaining two studies [37, 38].

### Duration of intervention

The duration of individual sessions ranged from 30 to 90 min, while the duration of the intervention ranged from one to five months. Weekly session frequency was for most studies between three to five sessions with one study reporting six sessions [38].

### Comparators

One study did not have a control group, reporting pre- post-intervention outcome on a single group [57]. Four studies used active controls, three of which consisted of music and dance-based therapies of the same intensity and frequency as the intervention [41, 53, 55]. The remaining study used reading training (teaching strategies for reading) at the same frequency and intensity of the intervention [40]. Nine studies used treatment as usual (TAU) as the control condition [37–39, 42, 51, 52, 54, 56, 58]. There was variation in the nature of the TAU condition including psychopharmacological treatment, psychoeducation in addition to medicines, psychosocial support, rehabilitation, occupational rehabilitation, or recreational activities. One study used a waitlist for the control group in addition to psychopharmacological treatment [39].

### Outcomes

#### Primary

Cognitive outcomes were assessed and reported by all studies; however, there was significant variation in the tools used to assess cognitive domains. The most frequently assessed domains included attention, memory, executive function, cognitive flexibility, problem-solving, planning, processing speed, social cognition, and metacognition. Various memory domains, i.e., visual, verbal, spatial and working memory were assessed in the included studies. One study included a module on facial affect recognition to target social cognition [58].

All apart from one study [40] assessed functional outcomes, but one did not report the effect of CR on this outcome [57]. Frequently assessed areas of functioning included social and occupational functioning, with the most common assessment instruments being the Global Assessment of Functioning (GAF), World Health Organisation Disability Assessment Schedule 2.0 (WHODAS 2.0) and Clinical Global Impressions (CGI).

#### Secondary

Of the fourteen studies included in the review, two assessed for QoL [38, 42], both of which used WHO Quality of Life-Brief Version (WHOQOL-BREF).

### Adverse events

There were no adverse events attributable to CR, however, across studies, it was mentioned that participants dropped out due to symptom deterioration.

### Assessors

Cognitive outcomes were most frequently assessed by trained psychologists, followed by researchers or research assistants and psychiatric nurses.

### Efficacy

Among the studies included, 13 showed evidence of efficacy across various cognitive domains [37–42, 51–57]. Eleven studies reported standardised effect sizes ranging from 0.11 to 1.73 [37–42, 52–56]; the remaining two studies showed significant improvement on cognitive performance post-intervention, but did not report effect sizes [51, 57]. Notably, in the remaining study, both the intervention and the control groups deteriorated on cognitive outcomes [58].

Areas of cognition improved by CR were executive functioning (cognitive flexibility, inhibitory control, problem solving), memory, learning, social cognition, processing speed and attention. Three studies reported sustained improvements [37, 38, 41] (see Table 4 for detailed results).

**Table 4:** Results of Studies

Study	Cognition	Functioning and Quality of Life
Hegde et al., 2012	Post-intervention, CR performed significantly better in the following, mean(SE): Motor speed—CR had higher measures in ‘finger tapping right’, 46(1.5) compared to TAU 40.6(1.4) (F = 5.96, *p* = 0.019). EF: Verbal N-back 1, errors were lower, 0.7(0.2) compared to 1.4(0.2) (F = 5.12, *p* = 0.03); Verbal N-back 2, hits were higher 5.7(0.2) compared to 4.8(0.2) (F = 4.60, *p* = 0.03); Verbal N-back errors were lower, 4.2(0.3) compared to 5.2(0.2) (F = 5.76, *p* = 0.02); WCST-number of trials administered was lower 110.6(2.8) compared to 120.6(2.6) (F = 6.06, *p* = 0.017); WCST-conceptual level responses were higher 53.3(3.2) compared to 41.2(3.0) (F = 6.65, *p* = 0.013); and WCST-categories complete was higher 4.2(0.3) compared to 3.1(0.2) (F = 4.84, *p* = 0.03) GLM indicated significant group x time interaction in: Tower of London test (TNMM) (F = 3.21, post-intervention d = 0.21, at follow-up d = 0.89); WCST number of trials administered (F = 4.56, post-intervention d = 1.45, at follow-up d = 0.97); WCST errors (F = 7.85, post-intervention d = 1.44, at follow-up d = 1.34); WCST preservative responses (F = 4.17, post-intervention d = 1.08, at follow-up d = 0.99); WCST preservative errors (F = 3.78, post-intervention d = 1.16, at follow-up d = 1.03); WCST conceptual level responses (F = 4.17, post-intervention d = 1.32, at follow-up d = 1.46); and WCST number of categories completed (F = 7.43, post-intervention d = 1.47, at follow-up d = 1.58) Memory—Verbal Learning and Memory: AVLT trial scores were higher in the treatment group 11.4(0.4) compared to 10.1(0.4) (F = 4.68, *p* = 0.03). Visual Learning and Memory: CFT-copy scores were higher in the treatment group 33.9(0.7) compared to 31.2(0.7) (F = 5.50, *p* = 0.02) Attention—Divided Attention: GLM indicated significant group x time interaction for the ‘triads test’, (F = 5.16, post-intervention d = 0.88, at follow-up d = 0.96)	Post-intervention, the treatment group performed significantly better in the following domains: Global functioning post-intervention indicated improved performance on the WHODAS (F = 258.96, *p* < 0.001).
Lu et al., 2012	Post-intervention, CR improved significantly in these measures of the WCST, mean(SD) EF, WCST (problem solving, abstraction and cognitive flexibility), mean(SD) Total numbers of completed cards at baseline 86(11.5) and post-intervention 73(12.2), (t = 6.49, *p* < 0.001). Number of cards correctly categorised, at baseline 22.3(2.4), post-treatment 25.8(4.5), (t=-5.29, *p* < 0.001). Number of preservative errors 29.2(2.5) at baseline, post-intervention 25.0(4.0) (t = 7.02, *p* < 0.001). Number of random errors at baseline 37.2(4.6), post-intervention 30.7(6.3), (t = 6.10, *p* < 0.001). Number of categories completed at baseline 2.5(0.8) and post-intervention 3.5(1.1), (t=-5.47, *p* < 0.001). Compared to TAU, improvements in the CR group were significantly greater for all cognitive measures—Total number of completed cards (F = 8.46, *p* < 0.004), Number of cards correctly categorised (F = 24.54, *p* < 0.001), Number of preservative errors (F = 15.29, *p* < 0.001) and number of categories completed (F = 5.62, *p* = 0.019)	Social functioning, mean(SD) Post-intervention, treatment group and TAU improved significantly; improvements in the treatment group was not significantly higher than in TAU. SSSI score (treatment) at baseline were 20.2(1.5), and post-treatment 13.6(2.1), (t = 20.25, *p* < 0.001); (F = 3.33, *p* = 0.074).
Byrne et al., 2013	Post-intervention, CR group performed significantly better than baseline on the LNS (t = 2.51, *p* = 0.02) and the HKLT (t = 3.32, *p* = 0.005). (verbal WM and verbal learning and memory respectively) CR group improved post-intervention on Digit Span (t = 4.41, *p* = 0.001), Word Span (t = 2.36, *p* = 0.035), Pair Matching (t = 2.59, *p* = 0.02), Spatial WM (t = 2.89, *p* = 0.013), and Arithmetic (t = 3.18, *p* = 0.007). Digit Span: WM, attention & Cognitive Flexibility; Word span; verbal WM, attention and language processing). Compared to Control, CR performed significantly better on the LNS (F = 11.41, *p* = 0.002), (Standardised ES = 0.50) [verbal WM]	Global Functioning: (F = 8.37, *p* = 0.007), Social Functioning: (F = 14.04, *p* = 0.001), (ES not reported)
Pontes et al., 2013	Post-intervention, the treatment group significantly improved in the following, mean(SE) Processing Speed—TMT- A: Treatment and control improved significantly at the follow-up; CR, from 57.2(41.2) to 49.2(26.5) and Control from 59(34) to 40(21) (*p* = 0.003). Improvement in CR was significantly better (Cohen’s d = 0.821, *p* = 0.019). [PS] Inhibitory Control—TMT-B—CR improved significantly from baseline; CR [155.8(89.30) to 141.5(153.8)], while the control deteriorated [121.1(60) to 128.5(98.9)], (*p* = 0.045). Stroop Test Card II—Both groups improved significantly from baseline; CR [28.1(15.4) to 23.6(15.3)] and control [22.2(10.5) to 17.8(5.7)], (*p* = 0.001). Stroop Card III—CR showed greater improvement over time (Cohen’s d = 1.549, *p* = 0.036). EF—MWCST preservative errors: Both groups showed a significant improvement CR [8.1(9.6) to 3(2.9)] and Control [8(9.9) to 1.8(3.4)], (*p* = 0.046); and MWCST failure to maintain set. Control did significantly better than the CR group over time (d = 1.086, *p* = 0.010). Memory(long-term verbal)—Logical Memory II: The control did significantly better than the CR group over time (d = 1.618, *p* = 0.003). Visual reproduction II: At follow-up, there was a significant improvement within groups; CR [28.6(9.9) to 29.1(12)] and control [13.5(9.1) to 23.5(15.1)], (*p* = 0.072).	Not Measured
Byrne et al., 2015	No improvements in any domains, mean(SD) CR and control differed at the baseline HKLT performance; CR performed worse, CR: 18.70(6.60); C: 22.95(5.74), (F = 4.71, *p* = 0.036), (standardised ES = 0.53). The performance of both groups deteriorated post-intervention, with CR performing significantly worse than baseline, 15.85(3.91), (t = 2.39, *p* = 0.027). Post-intervention, both groups did not differ significantly in this domain (F = 2.81, *p* = 0.102).	Compared to the baseline, CR group had a significant improvement in social functioning (PSP), Baseline mean(SD): 55.95(5.77); post-intervention: 58.63(6.13), (t=-3.06, *p* = 0.006), (Standardised ES = 0.68). CR group also performed better than the control post-intervention (F = 1.85, *p* = 0.018).
Sharifi et al., 2016	EF. Mean(SD)—SOC: The mean initial thinking time improved from 1707.52(1754.67) to 1159.50(1500.76), (t = 2.36, *p* = 0.02). The mean ‘subsequent thinking time decreased from 854.57(1523.12) to 141.51(386.52), (t = 2.25, p = 0.03), and the problems solved in minimum moves increased from 6.90(1.37) to 7.80(1.47), (t=-2.43, p = 0.02). IED: Completed stage errors decreased from 10.05(5.08) to 7.00(3.15), (t = 3.40, p = 0.003). Completed stage trials decreased from 70.45(9.37) to 63.30(7.02), (t = 2.96, p = 0.008). EDS errors decreased from 2.95(2.68) to 1.20(1.47), (t = 2.88, p = 0.009). Pre-ED errors decreased from 6.45(3.58) to 4.55(1.64), (t = 2.28, p = 0.03). Total errors decreased from 10.95(5.08) to 7.00(3.15), (t = 3.40, p = 0.003) and total trials decreased from 70.45(9.37) to 63.30(7.02), (t = 2.96, p = 0.008). Memory (visual), mean(SD)—Mean errors to success decreased from 3.73(2.62) to 2.21(1.54), (t = 2.37, *p* = 0.02). Mean trials to success decreased from 2.10(0.62) to 1.71(0.42), (t = 2.28, *p* = 0.03). Number of patterns succeeded on increased from 7.70(0.73) to 8.00(0), (t=-1.83, *p* = 0.08). Stages completed increased from 7.85(0.37) to 8.00(0), (t=-1.83, *p* = 0.08). Total errors decreased from 28.70(18.94) to 17.75(12.31), (t = 2.34, *p* = 0.03), and total trials decreased from 16.35(4.15) to 13.75(3.34), (t = 2.18, *p* = 0.04). While the mean of the first trial memory score improved from 15.40(4.38) to 17.10(4.23), the change was not significant (t=-1.35, *p* = 0.19). Similarly, stages completed on first trial improved from 4.90(1.07) to 5.50(1.00), but the change was not statistically significant (t=-1.55, *p* = 0.13). Attention, mean(SD)—Choice Reaction Time—There were statistically significant results in the following sub-scales: Percent correct trial improved from 98.05(2.80) to 99.40(0.99), (t=-2.52, *p* = 0.02). Mean correct latency decreased from 555.12(146.43) to 484.70(115.15), (t = 2.08, *p* = 0.05). The remaining subscales of the Choice Reaction time did not show significant changes: percent omission trials (t = 1.00, *p* = 0.32), percent omission trials (t = 0.94, *p* = 0.35), total commission errors (t = 1.08, *p* = 0.29) and total omission errors (t = 0.94, *p* = 0.35). SST—There were no significant changes in any of the subscales of SST. ‘Direction errors on stop and go trials’ (t = 1.21, p = 0.23); ‘proportion of successful stops’ (t = 0.67, p = 0.50); ‘median correct RT on GO trials’, (t = 0.70, p = 0.49); ‘SSD (50%) (last half)’ (t=-0.25) and SSRT (last half)’ (t = 1.51, p = 0.14). (ES not reported)	No significant change post intervention (ES not reported)
Tan et al., 2016	Mean (SD) Post-intervention, CR significantly better than baseline on the TMT-A [82.95(35.12) to 61.81(28.87), *p* < 0.05], Stroop-colour circle [31.43(19.74) to 23.66(9.46), *p* < 0.05], Stroop-coloured word [33.73(22.35) to 22.80(11.36), *p* < 0.05], Stroop-word colour [52.82(20.12) to 46.52(14.17), *p* < 0.05], BVRT-correct [4.05(1.88) to 6.26(1.82), *p* < 0.05] and the BVRT-wrong [10.13(4.80) to 5.62(3.18), *p* < 0.05]. CR improved significantly on the following compared to control—Cognitive Flexibility: TMT-A: (F = 9.53, *p* = 0.003), (d = 0.651); VFT: (F = 9.186, *p* = 0.003), (d = 0.639); Stroop-coloured word (F = 2.81, *p* = 0.097), (d = 0.366); and Stroop-word colour: (F = 12.44, *p* = 0.001), (d = 0.742) Memory: BVRT-correct: (F = 20.92, *p* < 0.001), (d = 1.006); and BVRT-wrong: (F = 17.58, *p* < 0.001), (d = 0.912)	Mean (SD) Social functioning: post-intervention, the treatment group improved significantly on the ‘NOSIE total’ [155.51(30.41) to 161.78(20.20), *p* < 0.05] (d = 0.465) and the ‘NOSIE general negative’ [24.81(16.18) to 22.32(10.92), *p* < 0.05] (d = 0.440)
Tan et al., 2020	MCCB total score favoured CR (F = 5.62, *p* = 0.02) ES: 0.27(0.04–0.47). Social Cognition—MSCEIT favoured CR (F = 4.55, *p* = 0.03) ES: 0.24(0.02–0.47). WM—Spatial Span Test favoured CR (F = 9.36, *p* < 0.001) ES: 0.32(0.11–0.53). Verbal Learning—HVLT-R favoured CR (F = 4.13, *p* = 0.04) ES: 0.22(0.01–0.43). Verbal WM—Digit Span test favoured CR (F = 8.84, *p* < 0.001) ES: 0.38(0.13–0.63); and EF—WCST favoured CR (F = 13.5, *p* < 0.001) ES: 0.42(0.20–0.65).	Social functioning—control group improved significantly more than the CR in the NOSIE negative factors at follow-up. [ES= -0.13(-0.33-0.11)]
Zhu et al., 2020	Compared to control, CR improved significantly post-intervention: MCCB total score ES: 0.35(0.01, 0.69), (F = 4.21, *p* = 0.04); Social Cognition MSCEIT ES: 0.31(0.01, 0.62), (F = 4.08, *p* = 0.05)	Compared to control, treatment improved significantly post-intervention: PSP ES: 0.44(0.13, 0.75), (F = 7.86, *p* = 0.01).
Zhu et al., 2021	Post-intervention, both CRT and CCRT improved significantly compared to the AC MCCB total score (F = 5.90, *p* = 0.003) (ES: CRT = 0.34, CCRT = 0.25); WM Spatial span test (F = 9.71, *p* < 0.001) (ES: CRT = 0.26, CCRT = 0.40); and Visual learning: BVMT-R (F = 6.75, *p* = 0.001) (ES: CRT = 0.38, CCRT = 0.24) CRT group improved significantly compared to the AC in the following: PS—TMT-A (F = 7.96, *p* < 0.001) (ES = 0.34); Symbol Coding test (F-8.17, *p* < 0.001) (ES = 0.29); and Reasoning and Problem solving: Mazes test: (F = 3.94, *p* = 0.001) (ES = 0.28). CCRT group improved significantly compared to the AC in the following: EF—WCST: (F = 7.81, *p* < 0.001) (ES = 0.34) and Social Cognition MSCEIT: (F = 5.05, *p* = 0.007) (ES = 0.11)	No Significant effects
Hatami et al., 2021	CR improved significantly more than control in the following post-treatment: Visual memory, mean(SD)—PAL first trial memory score: The CR performed significantly better post-treatment [15.6(4.6) to 18.1(4.4)], (F = 9.152, *p* = 0.004, Cohen’s d = 0.40); PAL Mean errors to success: [3.7(2.7) to 2.3(2.5)], (F = 6.991, *p* = 0.011, Cohen’s d = 0.14); Stages completed on first trial: (F = 7.155, *p* = 0.01, Cohen’s d = 0.71); and PAL total errors: [26.8(17.6) to 17.6(16.8)], (F = 5.730, *p* = 0.02, Cohen’s d = 0.53)	CR improved significantly more than control post-treatment: GAF: [6.5(1) to 6.8(0.87)], (Z=-2.125, *p* = 0.034); CGI: [2.1(0.86) to 1.8(0.77)], (Z=-2.119, *p* = 0.034). WHO QoL-Brief: [2.4(0.93) to 2.5(1)], (F = 5.239, *p* = 0.031). (ES not reported)
Moun et al., 2022	Post-intervention, CR had a significantly larger mean difference in the following: Verbal Memory: Mean improvement, CR [1.64(± 3.55)], control [0.07(± 1.31)] [t = 2.20(*p* = 0.034)] (d = 0.5). WM and Attention—Digit sequencing: Mean improvement, CR [0.64(± 1.34)], control, [-0.07(± 1.28)] [t = 2.05(*p* = 0.045)] (d = 0.4). Attention and Co-ordination—Token Motor: Mean improvement, CR [2.71(± 5.31)], control, [-0.07(± 1.96)] [t = 2.60(*p* = 0.013)] (d = 0.64). EF—Verbal fluency: Mean improvement, CR [0.82(± 1.51)], control [0.07(± 1.19)] [t = 2.08(*p* = 0.043)] (d = 0.69). Tower on London: Mean improvement, CR [1.82(± 4.00)], control, [-0.03(± 1.32)] [t = 2.33(*p* = 0.026)] (d = 0.64). PS—Symbol Coding: Mean improvement, CR [0.70(± 1.54)], control [-0.03(± 1.24)] [t = 2.02(*p* = 0.048)] (d = 0.6)	CR had a significantly larger mean difference in the following domains: Disability and functioning—WHODAS 2.0—Mean difference, treatment [-2.60(± 3.95)] control [0.06(± 0.52)] [t=-3.37(*p* = 0.002)]. WHOQOL-BREF—Mean difference, treatment [3.79(± 6.77)], control [0.10(± 0.31)] [t = 2.87(*p* = 0.008)] (ES not reported)
Singh et al., 2023	Post-intervention, CR improved significantly on the following: CR did significantly better than the control on the Verbal Fluency-COWA (Average new words): T [8.53(2.8) to 10.26(3.2)] and C [6.97(2.4) to 7.25(2.5)]; (F = 5.51, *p* = 0.036) (η² = 0.17). (EF) The CR significantly improved more over time than the control in the following: PS—T [322.06(92.3) to 269(65.9)] and C [349.28(138.5) to 346.57(109)], (F = 4.65, *p* = 0.04) (η² = 0.147). Sustained attention: Digit vigilance(time): T [707.26(165.1) to 568(154.8)] and C [651.35(154.7) to 625.85(179.7)], (F = 6.16, *p* = 0.02) (η² = 0.186). Verbal memory and learning—Auditory verbal learning test: Immediate recall: T [8.26(3.2) to 11.4(3.29)] and C [8.92(2.75) to 9.64(2.4)], (F = 7.70, *p* = 0.01) (η²=0.22); Delayed recall: T [8.4(2.8) to 10.66(2.76)] and C [8.28(2.58) to 8.64(2.4)], (F = 7.73, *p* = 0.01) (η²=0.22). Visual learning and memory: Immediate recall: T [14.2(6.3) to 21.26(6.14)] and C [16.96(8.04) to 18.75(8.3)], (F = 6.85, *p* = 0.014) (η² = 0.202); Delayed recall: T [12.06(6) to 18.13(7.25)] and C [13.92(9.54) to 15.42(7.7)], (F = 4.00, *p* = 0.055) (η² = 0.13)	The treatment group significantly improved more over time than the control in the following: Functioning: Treatment group did better on the IADL, T [4.13(1.12) to 5.06(0.96)] and C [4.28(1.32) to 4.78(1.18)], (F = 4.353, *p* = 0.047), (η² =0.139)
Deste et al., 2023	Compared TAU, post-intervention, CRT did significantly better on the following: WM: TMT-B (F = 17.22, *p* < 0.001), (Cohen’s D = 0.67); WAIS-R digit symbol coding (F = 10.33, *p* = 0.003), (Cohen’s D = 0.94). CF: WAIS-R digit span backword (F = 17.03, *p* < 0.001), (Cohen’s D = 1.73). PS: TMT-A (F = 11.87, *p* = 0.002), (Cohen’s D = 0.58); EF: TMT-B-A (8.08, *p* = 0.008), (Cohen’s D = 0.61). Verbal learning & Memory: RAVLT (12.12, *p* = 0.002), (Cohen’s D = 0.65)	Psychosocial functioning—GAF: No significant improvement when compared to control

TAU, treatment as usual; SE, standard errors; EF, executive function; WCST, Wisconsin Card Sorting Task; AVLT, Auditor Verbal Learning Task; CFT, Complex Figure Test; WHODAS, WHO Disability Assessment Schedule; SD, standard deviation; SSSI, Scale of Social Skills of Chronic Schizophrenia Patients; LNS, Letter Number Sequencing; HKLT, Hong Kong List Learning Test; WM, working memory; TMT-A, Trail-Making-Test-A; TMT-B, Trail Making Test-B; TMT B_A, Trail Making Test B-A; MWCST, modified Wisconsin card sorting test; SOC, Stocking of Cambridge; IED, intra-extra dimensional task; SST, stop signal task; BVRT, Benton Visual Retention Test; NOSIE, Nurse’s Observation Scale for Inpatient Evaluation; MCCB, MATRICS Consensus Cognitive battery; HVLT-R, Hopkins Verbal Learning Test-revised; NAB-Mazes, Neuropsychological Assessment Battery-Mazes; MSCEIT, Mayer-Salovery-Caruso Emotional Intelligence Test; PS, Processing Speed; CRT, Cognitive Remediation Therapy; CCRT, Computerised Cognitive Remediation Therapy; PAL, Paired Associate Learning; GAF, Global Assessment of Functioning; WHOQOL-BREF, World Health Organisation Quality of Life-Brief Version; RAVLT, Rey Auditory Verbal Learning Test; T, treatment group; C, control group; AC, active control; GLM, Generalised Linear Models and η², eta square

For attention, most studies reported small to moderate improvements [38, 53, 56], with one reporting large improvements [37]. Regarding executive functioning, four studies reported large improvements [37, 39, 40, 56], three reported moderate improvements [41, 52, 53], and two reported small to moderate improvements [38, 55]. Two studies reported moderate improvements in social cognition [41, 54], and one reported small improvement [55]. For processing speed, three studies reported moderate improvements [38, 39, 55], while two reported large improvements [40, 56]. Three studies reported large improvements in memory [40, 53, 56], and two reported moderate improvements [38, 42]. Lastly, improvements in learning ranged from large [56] to medium [39, 41, 55].

Findings related to functioning showed considerable variability; five studies reported effect sizes ranging from − 0.13 to 0.68 [38, 53, 56, 58] and four studies reported improvements, but did not report the effect sizes [37, 38, 42, 52]. Both control and intervention groups improved in one study [51], three reported no significant improvement post-intervention [39, 55, 57], and one study did not measure functioning [56] (see Table 4).

Two studies assessed for QoL, both showing significantly but moderated improved measures at follow-up (F = 5.239, *p* = 0.031; t = 2.87, *p* = 0.008), irrespective of the differing settings, delivery agent and mode of intervention delivery [38, 42].

### Efficacy across mode, agent and delivery setting

There were no significant differences in efficacy between computerised and manualised interventions, as both approaches yielded evidence of therapeutic benefit. Similarly, there was no observable difference between the seven studies delivering the intervention in groups [40, 41, 52–55, 58] and those delivering it individually [37–39, 42, 51, 56, 57]. There were no differences in efficacy across delivery agents, i.e., specialists [40, 41, 51, 53–56], non-specialists [39, 42, 52, 57, 58] and caregivers [37, 38]. Lastly, there were no differences in the efficacy of interventions towards cognitive or functional outcomes based on the place of delivery.

### Quality appraisal

Five randomised studies had a low risk of bias across all domains [40, 41, 53–55], while the remaining studies showed either a high risk [37, 38, 51, 52] or some concerns [42, 56]. Two studies had some concerns overall [42, 56], with both rated as concerning on domain 2 (deviations from intended intervention) and one in domain 1 (randomisation process) [42], the other in domain 5 (selection of reported results) [56]. Four had high concerning rating overall [37, 38, 51, 52] with domain 5 having lower ratings for all studies, and two studies had low rating for domain 4 (measurement of outcome) [38, 52]. Domain 1 [51], 2 [51], and 3 (missing outcome data) [38] were also problematic in some studies (See Figure S1, Online Resource 5). The three non-randomized studies had either serious [57, 58] or moderate overall bias [39], with concerns in domains such as confounding, outcome measurement, and participant selection (See Figure S2, Online Resource 5).

## Discussion

This systematic review examined the evidence of CR for people with psychosis who reside in LAMICs. It included and synthesised the results of fourteen studies conducted in different contexts.

This review confirms that CR can be a useful approach to reduce cognitive difficulties and improve functional outcomes and QoL, with effect sizes ranging significantly but overall suggesting benefits in line with evidence from high-income countries [19, 22]. The wide range of measures used made comparison difficult, and many studies did not comment on whether the measure they used was culturally validated and adapted to the context. Considering the range of approaches used, the different contexts, and the various measures employed, it is significant to observe a common trend suggesting the usefulness of this approach, perhaps indicating that there is more flexibility than previously thought on the way this approach could be delivered.

Irrespective of the method of intervention delivery—computerised or manualised formats, studies included in this review demonstrate evidence of benefit to cognitive, functional, and QoL outcomes. This finding is consistent with broader research, primarily based in high-income countries [19, 22]. Implementing CR via a manualised approach may have advantages in LAMICs due to inequitable access to technology [59, 60], low digital literacy [35, 61–63] and scarcity of resources. Manualised approaches may circumvent these barriers. However, one study within this review explored intervention delivery via tablets and found it improved cognitive and functional outcomes [56]. This, in contrast, highlights the potential of leveraging low-cost mobile health delivery modalities to facilitate CR uptake in resource-poor settings. There is an ongoing debate globally on the most efficient and easy-to-implement modality for CR delivery, and evidence is beginning to emerge on comparative advantages and equivalence of different approaches [64].

The efficacy of the intervention delivered did not seem to vary by setting. Studies in this review delivered the intervention in varied settings (i.e., homes, in-patient, and outpatient settings), catering to patients with differing levels of need and access to help available. People with mental illnesses face significant stigma, which is a barrier to help-seeking [65, 66], influencing both the person living with mental ill-health and the caregivers [67–69]. Moreover, people with psychosis are at an increased risk of stigma and violation of basic rights [70, 71]; this complicates management and reduces QoL. Home-based CR or CR linked with wider community health services might be a promising alternative to reduce the potential role stigma may have on attending interventions in mainstream clinical settings. The structure of family support and reliance on religious/traditional places of healing is an existing resource in LAMIC settings, which can be mobilised to facilitate culturally sensitive CR delivery [72, 73]. Evidence suggests that CR delivered in conjunction with other psychiatric rehabilitative services is more effective [19, 74]; in line with this, there is potential for integrating CR with existing community services in LAMICs. This also relates to WHO’s mhGAP intervention guidelines for psychoses, which highlight the importance of leveraging existing community resources to support people with severe mental health needs [75].

Interventions administered by either non-specialists or specialist practitioners demonstrated comparable efficacy on both cognitive, QoL and functional outcomes. This has significant implications for LAMIC settings, and resource-poor settings more broadly, where a dearth of resources and skewed ratios of specialist providers to patients are barriers to providing evidence-based mental health care [76]. Public health efforts often prioritise the management of infectious diseases [77, 78], diverting limited resources away from mental health services; cognitive outcomes and QoL for individuals with psychosis frequently remain neglected. In wider mental health research, there is robust evidence within LAMICs of task-shifting intervention delivery by lay health workers (LHWs), who, supervised by specialists, have successfully delivered mental health interventions using existing community resources [79, 80]. This may be a promising avenue to focus future research efforts.

This review has limitations. Two studies in languages other than English were excluded during screening due to poor translation quality. This may introduce a degree of publication bias limiting our findings only to studies available in English. Further, full-text documents from seven potentially eligible studies conducted in China could not be retrieved due to a lack of access to local databases. Lastly limitations of the studies considered, including the wide range of outcomes considered, lack of follow-up and fidelity and acceptability data, further limit the generalisability of our results.

There are some recommendations emerging from our review. In this field, there is an appreciation that measures translation have significant limitations, and careful adaptation is needed before cognitive measures can be used meaningfully. Measure adaptation to low-income settings should consider cultural, socio-economic, and literacy differences. There are emerging examples and good practice in this sense, such as the Ethiopian Cognitive Assessment battery in Schizophrenia (ECAS) [81]. Overall, the studies considered had significant shortfalls in terms of methodological rigour. Robust, large and well-conducted trials often require a high level of resources and expertise that may not yet be present in many LAMICs. Collaborative endeavours, sharing resources, and clear guidelines may need to be in place before the evidence quality can improve.

Ten of the 14 identified studies were conducted in upper‑middle‑income countries [40–42, 51–55, 57, 58], underscoring a substantial evidence gap regarding CR interventions for people with psychosis in low‑ and lower‑middle‑income settings. Future research should prioritise studies in low‑income countries to address this imbalance and strengthen evidence in this area.

Future efforts should be directed towards exploring directly the acceptability of CR within LAMIC settings. Culturally valid measurement instruments should be employed to ensure cognitive and functional outcomes accuracy, and interventions should be contextually appropriate. There is an urgent need to conduct studies using pragmatic and scalable methods of CR deployment. Many of the studies reviewed in this study take place in highly structured clinical settings (e.g., inpatient facilities). These adaptations need to be sensitive to the setting but also allow CR to become available to more individuals.

CR is still scarcely available globally, and this poor availability may have different reasons in different settings and countries. In LAMICs, clinicians may not have sufficient training to recognise, assess and manage cognitive difficulties. For this to change, we may need to consider ways that are culturally and contextually sensitive to promote change. Caregiver involvement and understanding of cognitive difficulties may be a crucial first step, along with the promotion of regular assessment and support, particularly for those presenting with the most severe cognitive difficulties.

## Supplementary Information

Below is the link to the electronic supplementary material.


Supplementary Material 1



Supplementary Material 2



Supplementary Material 3



Supplementary Material 4



Supplementary Material 5


## Data Availability

No datasets were generated or analysed during the current study.
