# Supplementary material for: Cognitive remediation therapy in low-and-middle-income countries (LAMICs) for people with psychosis: a systematic review of its efficacy on functional and cognitive outcomes
Source: Soc Psychiatry Psychiatr Epidemiol. 2026 Jun 11;61(8):1361–86. doi: 10.1007/s00127-026-03136-x (PMC13522050; doi:10.1007/s00127-026-03136-x)
Supplement: Supplementary file 2 [file 127_2026_3136_MOESM2_ESM.docx]

Online Resource 2: Search Strategies

*Social Psychiatry and Psychiatric Epidemiology*

**Cognitive Remediation Therapy in Low-and-Middle-Income Countries (LAMICs): A systematic review of its efficacy on functional and cognitive outcomes**

Yamini, Vig ^1,2,3^; Yohannes Gebreegziabhere 4,5; Charlotte, Hanlon 4,6; Matteo, Cella1,7.

1 - Institute of Psychiatry, Psychology and Neuroscience, King's College London, London, UK.

2 - Centre for Global Mental Health, London School of Hygiene and Tropical Medicine, London, UK.

3 - Addictions and Related Research Group (ARG), Sangath, Porvorim, Goa, India. Email: [yaminivig16@gmail.com](mailto:yaminivig16@gmail.com). ORCID: 0009-0001-9288-5939

4 - Department of Psychiatry, College of Health Sciences, Addis Ababa University, Addis Ababa, Ethiopia.

5 - Department of Nursing, College of Health Sciences, Debre Berhan University, Debre Berhan, Ethiopia. Email: [yohannes36@gmail.com](mailto:yohannes36@gmail.com).

6 - Division of Psychiatry, Centre for Clinical Brain Sciences. The University of Edinburgh, Edinburgh, Scotland, UK; and Department of Psychiatry, School of Medicine, College of Health Sciences, Addis Ababa University, Addis Ababa, Ethiopia. Email: [chanlon@ed.ac.uk](mailto:chanlon@ed.ac.uk). ORCID: 0000-0002-7937-3226

7 - South London and Maudsley NHS Foundation Trust, London, UK. 0000-0002-5701-0336

Search Strategies (Global Index Medicus, OVID MEDLINE(R), OVID Embase, OVID APA PsycINFO, OVID Global Health, Web of Science, and SCOPUS)

Table S1. Database Search Strategies

| Global Index Medicus Search Strategy | |
| --- | --- |
| Search Strategy | tw:((mh:( f04.754.137.365 OR e02.831.477.250 ) OR tw:("cognitive remediation" OR "cognitive rehabilitation" OR "cognitive training" OR "cognitive retraining" OR "cognitive enhancement" OR "brain training" )) AND (mh:( f03 OR f03.600.150.500 OR f03.700.150 OR f03.700.675 OR f03.700.450 OR f03.700.750.650 OR f03.700.750.600 OR f03.700.750.350 OR f03.700.750.300) OR tw:("bipolar disorder with psychotic" OR "bipolar disorder with psychosis" OR "bipolar disorder with psychoses" OR "bipolar affective disorder with psychotic" OR "bipolar affective disorder with psychosis" OR "bipolar affective disorder with psychoses" OR "manic depression with psychosis" OR "manic depression with psychoses" OR "manic depression with psychotic" OR "manic depressive illness with psychosis" OR "manic depressive illness with psychoses" OR "manic depressive illness with psychotic" OR "bipolar depression with psychotic" OR "bipolar depression with psychosis" OR "bipolar depression with psychoses" OR "bipolar depression with psychotic" OR "bipolar mood disorder with psychotic" OR "bipolar mood disorder with psychosis" OR "bipolar mood disorder with psychoses" OR "manic psychosis" OR "manic psychoses" OR "mania with psychosis" OR "mania with psychoses" OR "affective psychosis" OR "affective psychoses" OR "affective disorder with psychosis" OR "affective disorder with psychoses" OR "affective disorder with psychotic" OR "mood disorder with psychosis" OR "mood disorder with psychoses" OR "BPAD with psychosis" OR "BPAD with psychoses" OR "BPAD with psychotic" OR "BD with psychosis" OR "BD with psychoses" OR "BD with psychotic" OR "mania with psychosis" OR "mania with psychoses" OR "mania with psychotic" ) OR ("depressive psychosis" OR "depressive psychoses" OR "depression with psychosis" OR "depression with psychoses" OR "depression with psychotic" OR "psychotic depression" OR "major depressive disorder with psychosis" OR "major depressive disorder with psychoses" OR "major depressive disorder with psychotic" OR "major depression with psychosis" OR "major depression with psychoses" OR "major depression with psychotic" OR "MDD with psychosis" OR "MDD with psychoses" OR "MDD with psychotic") OR ("severe mental" OR "serious mental" OR "SMD" OR "SMI") OR (schizophren* OR schizoaffective OR “schizo-affective” OR schizotyp* OR psychosis OR psychoses OR psychot*))) |

| Search Strategies OVID Databases | | | |
| --- | --- | --- | --- |
| Databases | Population/Exposure | Intervention | Setting |
| OVID MEDLINE(R) | "schizophrenia spectrum and other psychotic disorders"/ or affective disorders, psychotic/ or paranoid disorders/ or psychotic disorders/ or exp schizophrenia/  OR  (bipolar dis* adj2 psychos#s) OR (bipolar adj2 psychos#s) OR (bipolar depress* adj2 psychos#s) OR affective psychos#s OR bipolar affective psychos#s OR (bipolar mood disorder adj2 psychos#s) OR (bipolar affective dis* adj2 psychos#s) OR manic psychos#s OR (mania adj2 psychos#s) OR (BD adj2 psychos#s) OR manic depress* psychos#s OR (mood disorders adj2 psychos#s) OR (bipolar dis* adj2 psychot*) OR (bipolar adj2 psychot*) OR (bipolar depress* adj2 psychot*) OR (manic depress* adj2 psychot*) OR affective psychot* OR (affective disorder adj2 psychot*) OR (bipolar mood disorder adj2 psychot*) OR (bipolar affective dis* adj2 psychot*) OR (mania adj2 psychot*) OR (BPAD adj2 psychot*) OR (BD adj2 psychot*) OR (mood disorders adj2 psychot*) OR (manic depress* adj2 psychot*) OR (depress* adj2 psychos#s) OR (major depress* adj2 psychos#s) OR (MDD adj2 psychos#s) OR (depress* adj2 psychot*) OR (major depress* adj2 psychot*) OR (MDD adj2 psychot*) OR psychot* depress* OR schizophren* OR schizotyp* OR schizo?affect* OR psychos#s OR psychot* OR severe mental illnes* OR severe mental dis* OR serious mental illnes* OR serious mental dis* | exp Cognitive Remediation/ OR exp Cognitive Training/ OR cognitive remediation OR cognitive rehab* OR cognitive training OR cognitive retraining OR cognitive enhancement OR brain training | (afghan* or africa* or albania* or algeria* or angola* or antigua* or barbuda* or argentin* or armenia* or aruba* or azerbaijan* or bahrain* or bangladesh* or bengal* or bangal* or barbados* or barbadian* or bajan or bajans or belarus* or belorus* or byelarus* or byelorus* or belize* or benin* or dahomey or bhutan* or bolivia* or bosnia* or herzegovin* or botswan* or batswan* or bechuanaland* or brazil* or brasil* or bulgaria* or burkina* or burkinese* or upper volta* or burundi* or urundi* or cabo verde* or cape verde* or cambodia* or kampuchea* or khmer* or cameroon* or cameroun* or ubangi shari* or chad* or chile* or china* or chinese or colombia* or comoro* or comore* or comorian* or mayotte* or congo* or zaire* or costa rica* or "cote d'ivoir*" or "cote d' ivoir*" or cote divoir* or cote d ivoir* or ivory coast* or ivorian* or croatia* or cuba or cuban or cubans or "cuba's" or cyprus* or cypriot* or czech* or djibouti* or french somaliland* or dominica* or ecuador* or egypt* or united arab republic* or el salvador* or salvadoran* or guinea* or equatoguinea* or eritrea* or estonia* or eswatini* or swaziland* or swazi* or swati* or ethiopia* or fiji* or gabon* or gabonese* or gabonaise* or gambia* or ((georgia or georgian or georgians) not (atlanta or california or florida)) or ghana* or gibraltar* or greece* or greek* or grecian* or grenada* or grenadian* or guam* or guatemala* or guyana* or guiana* or guyanese* or haiti* or hispaniola* or hondura* or hungary* or hungarian* or india* or indonesia* or iran* or iraq* or isle of man* or jamaica* or jordan* or kazakh* or kenya* or karabati* or korea* or kosovo* or kosova* or kyrgyz* or kirgiz* or kirghiz* or laos or lao or laotian* or latvia* or lebanon* or lebanese* or lesotho* or lesothan* or lesothonian* or basutoland* or mosotho* or basotho* or liberia* or libya* or jamahiriya* or lithuania* or macedonia* or madagasca* or malagasy* or malawi* or nyasaland* or malaysia* or malay* federation or maldives* or maldivian* or indian ocean or mali or malian* or "mali's" or malta or maltese* or "malta's" or micronesia* or marshallese* or kiribati* or marshall island* or nauru or nauran or nauruans or "naurian's" or mariana or marianas or palau or paluan* or tuvalu* or mauritania* or mauritan* or mauritius* or mexico* or mexican* or moldova* or moldovia* or mongol* or montenegr* or morocco* or moroccan* or ifni or mozambique* or mozambican* or myanmar* or burma* or burmese or namibia* or nepal* or new caledonia* or netherlands antill* or nicaragua* or niger* or oman or omani or omanis or "oman's" or pakistan* or palestin* or gaza* or west bank* or panama* or paraguay* or peru or peruvian* or "peru's" or philippine* or philipine* or phillipine* or phillippine* or filipino* or filipina* or poland* or polish or pole or poles or portugal* or portuguese or puerto ric* or romania* or russia* or ussr* or soviet* or rwanda* or rwandese or ruanda* or ruandese or samoa* or navigator island* or pacific island* or polynesia* or sao tome* or santomean* or saudi arabia* or saudi or saudis or senegal* or serbia* or seychell* or sierra leone* or slovak* or sloven* or melanesia* or solomon island* or norfolk island* or somali* or sri lanka* or ceylon* or saint kitts or st kitts or kittian* or nevisian* or saint lucia* or st lucia* or saint vincent* or st vincent* or vincentian* or grenadine* or sudan* or surinam* or syria* or tajik* or tadjik* or tadzhik* or tanzania* or tanganyika* or thai* or timor leste* or east timor* or timorese* or togo or togoles* or "togo's" or tonga* or trinidad* or tobago* or tunisia* or turkiy* or turkey* or turk or turks or turkish or turkmen* or uganda* or ukrain* or uruguay* or uzbek* or vanuatu* or new hebrides* or venezuela* or vietnam* or viet nam* or yemen* or yugoslav* or zambia* or zimbabwe* or rhodesia* or arab* countr* or middle east* or global south or sahara* or subsahara* or magreb* or maghrib* or west indies* or caribbean* or central america* or latin america* or south america* or asia central or central asia* or asia northern or north asia* or northern asia* or asia southeastern or southeastern asia* or south eastern asia* or southeast asia* or south east asia* or asia western or west asia* or western asia* or europe eastern or east europe* or eastern europe* or developing countr* or developing nation* or developing population* or developing world or less developed countr* or less developed nation* or less developed world or lesser developed countr* or lesser developed nation* or lesser developed world or under developed countr* or under developed nation* or under developed world or underdeveloped countr* or underdeveloped nation* or underdeveloped world or middle income countr* or middle income nation* or middle income population* or low income countr* or low income nation* or low income population* or lower income countr* or lower income nation* or lower income population* or underserved countr* or underserved nation* or underserved population* or under served population* or under served nation* or under served population* or deprived countr* or deprived population* or high burden countr* or high burden nation* or countdown countr* or countdown nation* or poor countr* or poor nation* or poor population* or poor world or poorer countr* or poorer nation* or poorer population* or poorer world or developing econom* or less developed econom* or underdeveloped econom* or under developed econom* or middle income econom* or low income econom* or lower income econom* or low gdp or low gnp or low gross domestic or low gross national or lower gdp or lower gnp or lower gross domestic or lower gross national or lmic or lmics or third world or lami countr* or transitional countr* or emerging econom* or emerging nation*).ti,ab,hw,kf. |
| OVID Embase | exp psychosis/ OR exp affective psychosis/ OR exp schizophrenia spectrum disorder/  OR  (bipolar dis* adj2 psychos#s) OR (bipolar adj2 psychos#s) OR (bipolar depress* adj2 psychos#s) OR affective psychos#s OR bipolar affective psychos#s OR (bipolar mood disorder adj2 psychos#s) OR (bipolar affective dis* adj2 psychos#s) OR (bipolar affective dis* adj2 psychos#s) OR manic psychos#s OR (mania adj2 psychos#s) OR (BD adj2 psychos#s) OR manic depress* psychos#s OR (mood disorders adj2 psychos#s) OR (bipolar dis* adj2 psychot*) OR (bipolar adj2 psychot*) OR (bipolar depress* adj2 psychot*) OR (manic depress* adj2 psychot*) OR affective psychot* OR (affective disorder adj2 psychot*) OR (bipolar mood disorder adj2 psychot*) OR (bipolar affective dis* adj2 psychot*) OR (mania adj2 psychot*) OR (BPAD adj2 psychot*) OR (BD adj2 psychot*) OR (mood disorders adj2 psychot*) OR (manic depress* adj2 psychot*) OR (depress* adj2 psychos#s) OR (major depress* adj2 psychos#s) OR (MDD adj2 psychos#s) OR (depress* adj2 psychot*) OR (major depress* adj2 psychot*) OR (MDD adj2 psychot*) OR psychot* depress* OR schizophren* OR schizotyp* OR schizo?affect* OR psychos#s OR psychot* OR severe mental illnes* OR severe mental dis* OR serious mental illnes* OR serious mental dis* OR | exp cognitive remediation therapy/ OR exp cognitive rehabilitation/  OR  cognitive remediation OR cognitive rehab* OR cognitive training OR cognitive retraining OR cognitive enhancement OR brain training | (afghan* or africa* or albania* or algeria* or angola* or antigua* or barbuda* or argentin* or armenia* or aruba* or azerbaijan* or bahrain* or bangladesh* or bengal* or bangal* or barbados* or barbadian* or bajan or bajans or belarus* or belorus* or byelarus* or byelorus* or belize* or benin* or dahomey or bhutan* or bolivia* or bosnia* or herzegovin* or botswan* or batswan* or bechuanaland* or brazil* or brasil* or bulgaria* or burkina* or burkinese* or upper volta* or burundi* or urundi* or cabo verde* or cape verde* or cambodia* or kampuchea* or khmer* or cameroon* or cameroun* or ubangi shari* or chad* or chile* or china* or chinese or colombia* or comoro* or comore* or comorian* or mayotte* or congo* or zaire* or costa rica* or "cote d'ivoir*" or "cote d' ivoir*" or cote divoir* or cote d ivoir* or ivory coast* or ivorian* or croatia* or cuba or cuban or cubans or "cuba's" or cyprus* or cypriot* or czech* or djibouti* or french somaliland* or dominica* or ecuador* or egypt* or united arab republic* or el salvador* or salvadoran* or guinea* or equatoguinea* or eritrea* or estonia* or eswatini* or swaziland* or swazi* or swati* or ethiopia* or fiji* or gabon* or gabonese* or gabonaise* or gambia* or ((georgia or georgian or georgians) not (atlanta or california or florida)) or ghana* or gibraltar* or greece* or greek* or grecian* or grenada* or grenadian* or guam* or guatemala* or guyana* or guiana* or guyanese* or haiti* or hispaniola* or hondura* or hungary* or hungarian* or india* or indonesia* or iran* or iraq* or isle of man* or jamaica* or jordan* or kazakh* or kenya* or karabati* or korea* or kosovo* or kosova* or kyrgyz* or kirgiz* or kirghiz* or laos or lao or laotian* or latvia* or lebanon* or lebanese* or lesotho* or lesothan* or lesothonian* or basutoland* or mosotho* or basotho* or liberia* or libya* or jamahiriya* or lithuania* or macedonia* or madagasca* or malagasy* or malawi* or nyasaland* or malaysia* or malay* federation or maldives* or maldivian* or indian ocean or mali or malian* or "mali's" or malta or maltese* or "malta's" or micronesia* or marshallese* or kiribati* or marshall island* or nauru or nauran or nauruans or "naurian's" or mariana or marianas or palau or paluan* or tuvalu* or mauritania* or mauritan* or mauritius* or mexico* or mexican* or moldova* or moldovia* or mongol* or montenegr* or morocco* or moroccan* or ifni or mozambique* or mozambican* or myanmar* or burma* or burmese or namibia* or nepal* or new caledonia* or netherlands antill* or nicaragua* or niger* or oman or omani or omanis or "oman's" or pakistan* or palestin* or gaza* or west bank* or panama* or paraguay* or peru or peruvian* or "peru's" or philippine* or philipine* or phillipine* or phillippine* or filipino* or filipina* or poland* or polish or pole or poles or portugal* or portuguese or puerto ric* or romania* or russia* or ussr* or soviet* or rwanda* or rwandese or ruanda* or ruandese or samoa* or navigator island* or pacific island* or polynesia* or sao tome* or santomean* or saudi arabia* or saudi or saudis or senegal* or serbia* or seychell* or sierra leone* or slovak* or sloven* or melanesia* or solomon island* or norfolk island* or somali* or sri lanka* or ceylon* or saint kitts or st kitts or kittian* or nevisian* or saint lucia* or st lucia* or saint vincent* or st vincent* or vincentian* or grenadine* or sudan* or surinam* or syria* or tajik* or tadjik* or tadzhik* or tanzania* or tanganyika* or thai* or timor leste* or east timor* or timorese* or togo or togoles* or "togo's" or tonga* or trinidad* or tobago* or tunisia* or turkiy* or turkey* or turk or turks or turkish or turkmen* or uganda* or ukrain* or uruguay* or uzbek* or vanuatu* or new hebrides* or venezuela* or vietnam* or viet nam* or yemen* or yugoslav* or zambia* or zimbabwe* or rhodesia* or arab* countr* or middle east* or global south or sahara* or subsahara* or magreb* or maghrib* or west indies* or caribbean* or central america* or latin america* or south america* or asia central or central asia* or asia northern or north asia* or northern asia* or asia southeastern or southeastern asia* or south eastern asia* or southeast asia* or south east asia* or asia western or west asia* or western asia* or europe eastern or east europe* or eastern europe* or developing countr* or developing nation* or developing population* or developing world or less developed countr* or less developed nation* or less developed world or lesser developed countr* or lesser developed nation* or lesser developed world or under developed countr* or under developed nation* or under developed world or underdeveloped countr* or underdeveloped nation* or underdeveloped world or middle income countr* or middle income nation* or middle income population* or low income countr* or low income nation* or low income population* or lower income countr* or lower income nation* or lower income population* or underserved countr* or underserved nation* or underserved population* or under served population* or under served nation* or under served population* or deprived countr* or deprived population* or high burden countr* or high burden nation* or countdown countr* or countdown nation* or poor countr* or poor nation* or poor population* or poor world or poorer countr* or poorer nation* or poorer population* or poorer world or developing econom* or less developed econom* or underdeveloped econom* or under developed econom* or middle income econom* or low income econom* or lower income econom* or low gdp or low gnp or low gross domestic or low gross national or lower gdp or lower gnp or lower gross domestic or lower gross national or lmic or lmics or third world or lami countr* or transitional countr* or emerging econom* or emerging nation*).ti,ab,hw,kf. |
| OVID APA PsychInfo | psychosis/ or affective psychosis/ or brief psychotic disorder/ or childhood onset psychosis/ or chronic psychosis/ or delusional disorder/ or paranoid psychosis/ or postpartum psychosis/ or exp schizophrenia/  OR  (bipolar dis* adj2 psychos#s) OR (bipolar adj2 psychos#s) OR (bipolar depress* adj2 psychos#s) OR affective psychos#s OR bipolar affective psychos#s OR (bipolar mood disorder adj2 psychos#s) OR (bipolar affective dis* adj2 psychos#s) OR manic psychos#s OR (mania adj2 psychos#s) OR (BD adj2 psychos#s) OR manic depress* psychos#s OR (mood disorders adj2 psychos#s) OR (bipolar dis* adj2 psychot*) OR (bipolar adj2 psychot*) OR (bipolar depress* adj2 psychot*) OR (manic depress* adj2 psychot*) OR affective psychot* OR (affective disorder adj2 psychot*) OR (bipolar mood disorder adj2 psychot*) OR (bipolar affective dis* adj2 psychot*) OR (mania adj2 psychot*) OR (mania adj2 psychot*) OR (BPAD adj2 psychot*) OR (BD adj2 psychot*) OR (mood disorders adj2 psychot*) OR (manic depress* adj2 psychot*) OR (depress* adj2 psychos#s) OR (major depress* adj2 psychos#s) OR (MDD adj2 psychos#s) OR (depress* adj2 psychot*) OR (major depress* adj2 psychot*) OR (MDD adj2 psychot*) OR psychot* depress* OR schizophren* OR schizotyp* OR schizo?affect* OR psychos#s OR psychot* OR severe mental illnes* OR severe mental dis* OR serious mental illnes* OR serious mental dis* | exp cognitive rehabilitation/ or exp cognitive remediation/ OR brain training/  OR  cognitive remediation OR cognitive rehab* OR cognitive training OR cognitive retraining OR brain training OR cognitive enhancement | (afghan* or africa* or albania* or algeria* or angola* or antigua* or barbuda* or argentin* or armenia* or aruba* or azerbaijan* or bahrain* or bangladesh* or bengal* or bangal* or barbados* or barbadian* or bajan or bajans or belarus* or belorus* or byelarus* or byelorus* or belize* or benin* or dahomey or bhutan* or bolivia* or bosnia* or herzegovin* or botswan* or batswan* or bechuanaland* or brazil* or brasil* or bulgaria* or burkina* or burkinese* or upper volta* or burundi* or urundi* or cabo verde* or cape verde* or cambodia* or kampuchea* or khmer* or cameroon* or cameroun* or ubangi shari* or chad* or chile* or china* or chinese or colombia* or comoro* or comore* or comorian* or mayotte* or congo* or zaire* or costa rica* or "cote d'ivoir*" or "cote d' ivoir*" or cote divoir* or cote d ivoir* or ivory coast* or ivorian* or croatia* or cuba or cuban or cubans or "cuba's" or cyprus* or cypriot* or czech* or djibouti* or french somaliland* or dominica* or ecuador* or egypt* or united arab republic* or el salvador* or salvadoran* or guinea* or equatoguinea* or eritrea* or estonia* or eswatini* or swaziland* or swazi* or swati* or ethiopia* or fiji* or gabon* or gabonese* or gabonaise* or gambia* or ((georgia or georgian or georgians) not (atlanta or california or florida)) or ghana* or gibraltar* or greece* or greek* or grecian* or grenada* or grenadian* or guam* or guatemala* or guyana* or guiana* or guyanese* or haiti* or hispaniola* or hondura* or hungary* or hungarian* or india* or indonesia* or iran* or iraq* or isle of man* or jamaica* or jordan* or kazakh* or kenya* or karabati* or korea* or kosovo* or kosova* or kyrgyz* or kirgiz* or kirghiz* or laos or lao or laotian* or latvia* or lebanon* or lebanese* or lesotho* or lesothan* or lesothonian* or basutoland* or mosotho* or basotho* or liberia* or libya* or jamahiriya* or lithuania* or macedonia* or madagasca* or malagasy* or malawi* or nyasaland* or malaysia* or malay* federation or maldives* or maldivian* or indian ocean or mali or malian* or "mali's" or malta or maltese* or "malta's" or micronesia* or marshallese* or kiribati* or marshall island* or nauru or nauran or nauruans or "naurian's" or mariana or marianas or palau or paluan* or tuvalu* or mauritania* or mauritan* or mauritius* or mexico* or mexican* or moldova* or moldovia* or mongol* or montenegr* or morocco* or moroccan* or ifni or mozambique* or mozambican* or myanmar* or burma* or burmese or namibia* or nepal* or new caledonia* or netherlands antill* or nicaragua* or niger* or oman or omani or omanis or "oman's" or pakistan* or palestin* or gaza* or west bank* or panama* or paraguay* or peru or peruvian* or "peru's" or philippine* or philipine* or phillipine* or phillippine* or filipino* or filipina* or poland* or polish or pole or poles or portugal* or portuguese or puerto ric* or romania* or russia* or ussr* or soviet* or rwanda* or rwandese or ruanda* or ruandese or samoa* or navigator island* or pacific island* or polynesia* or sao tome* or santomean* or saudi arabia* or saudi or saudis or senegal* or serbia* or seychell* or sierra leone* or slovak* or sloven* or melanesia* or solomon island* or norfolk island* or somali* or sri lanka* or ceylon* or saint kitts or st kitts or kittian* or nevisian* or saint lucia* or st lucia* or saint vincent* or st vincent* or vincentian* or grenadine* or sudan* or surinam* or syria* or tajik* or tadjik* or tadzhik* or tanzania* or tanganyika* or thai* or timor leste* or east timor* or timorese* or togo or togoles* or "togo's" or tonga* or trinidad* or tobago* or tunisia* or turkiy* or turkey* or turk or turks or turkish or turkmen* or uganda* or ukrain* or uruguay* or uzbek* or vanuatu* or new hebrides* or venezuela* or vietnam* or viet nam* or yemen* or yugoslav* or zambia* or zimbabwe* or rhodesia* or arab* countr* or middle east* or global south or sahara* or subsahara* or magreb* or maghrib* or west indies* or caribbean* or central america* or latin america* or south america* or asia central or central asia* or asia northern or north asia* or northern asia* or asia southeastern or southeastern asia* or south eastern asia* or southeast asia* or south east asia* or asia western or west asia* or western asia* or europe eastern or east europe* or eastern europe* or developing countr* or developing nation* or developing population* or developing world or less developed countr* or less developed nation* or less developed world or lesser developed countr* or lesser developed nation* or lesser developed world or under developed countr* or under developed nation* or under developed world or underdeveloped countr* or underdeveloped nation* or underdeveloped world or middle income countr* or middle income nation* or middle income population* or low income countr* or low income nation* or low income population* or lower income countr* or lower income nation* or lower income population* or underserved countr* or underserved nation* or underserved population* or under served population* or under served nation* or under served population* or deprived countr* or deprived population* or high burden countr* or high burden nation* or countdown countr* or countdown nation* or poor countr* or poor nation* or poor population* or poor world or poorer countr* or poorer nation* or poorer population* or poorer world or developing econom* or less developed econom* or underdeveloped econom* or under developed econom* or middle income econom* or low income econom* or lower income econom* or low gdp or low gnp or low gross domestic or low gross national or lower gdp or lower gnp or lower gross domestic or lower gross national or lmic or lmics or third world or lami countr* or transitional countr* or emerging econom* or emerging nation*).ti,ab,hw,id. |
| OVID Global Health | exp psychoses/ or exp schizophrenia/  OR  (bipolar dis* adj2 psychos#s) OR (bipolar adj2 psychos#s) OR (bipolar depress* adj2 psychos#s) OR affective psychos#s OR bipolar affective psychos#s OR (bipolar mood disorder adj2 psychos#s) OR (bipolar affective dis* adj2 psychos#s) OR manic psychos#s OR (mania adj2 psychos#s) OR (BD adj2 psychos#s) OR manic depress* psychos#s OR (mood disorders adj2 psychos#s) OR (bipolar dis* adj2 psychot*) OR (bipolar adj2 psychot*) OR (bipolar depress* adj2 psychot*) OR (manic depress* adj2 psychot*) OR affective psychot* OR (affective disorder adj2 psychot*) OR (bipolar mood disorder adj2 psychot*) OR (bipolar affective dis* adj2 psychot*) OR (mania adj2 psychot*) OR (BPAD adj2 psychot*) OR (BD adj2 psychot*) OR (mood disorders adj2 psychot*) OR (manic depress* adj2 psychot*) OR (depress* adj2 psychos#s) OR (major depress* adj2 psychos#s) OR (MDD adj2 psychos#s) OR (depress* adj2 psychot*) OR (major depress* adj2 psychot*) OR (MDD adj2 psychot*) OR psychot* depress* OR schizophren* OR schizotyp* OR schizo?affect* OR psychos#s OR psychot* OR severe mental illnes* OR severe mental dis* OR serious mental illnes* OR serious mental dis* | cognitive remediation OR cognitive rehab* OR cognitive training OR cognitive enhancement OR brain training | (afghan* or africa* or albania* or algeria* or angola* or antigua* or barbuda* or argentin* or armenia* or aruba* or azerbaijan* or bahrain* or bangladesh* or bengal* or bangal* or barbados* or barbadian* or bajan or bajans or belarus* or belorus* or byelarus* or byelorus* or belize* or benin* or dahomey or bhutan* or bolivia* or bosnia* or herzegovin* or botswan* or batswan* or bechuanaland* or brazil* or brasil* or bulgaria* or burkina* or burkinese* or upper volta* or burundi* or urundi* or cabo verde* or cape verde* or cambodia* or kampuchea* or khmer* or cameroon* or cameroun* or ubangi shari* or chad* or chile* or china* or chinese or colombia* or comoro* or comore* or comorian* or mayotte* or congo* or zaire* or costa rica* or "cote d'ivoir*" or "cote d' ivoir*" or cote divoir* or cote d ivoir* or ivory coast* or ivorian* or croatia* or cuba or cuban or cubans or "cuba's" or cyprus* or cypriot* or czech* or djibouti* or french somaliland* or dominica* or ecuador* or egypt* or united arab republic* or el salvador* or salvadoran* or guinea* or equatoguinea* or eritrea* or estonia* or eswatini* or swaziland* or swazi* or swati* or ethiopia* or fiji* or gabon* or gabonese* or gabonaise* or gambia* or ((georgia or georgian or georgians) not (atlanta or california or florida)) or ghana* or gibraltar* or greece* or greek* or grecian* or grenada* or grenadian* or guam* or guatemala* or guyana* or guiana* or guyanese* or haiti* or hispaniola* or hondura* or hungary* or hungarian* or india* or indonesia* or iran* or iraq* or isle of man* or jamaica* or jordan* or kazakh* or kenya* or karabati* or korea* or kosovo* or kosova* or kyrgyz* or kirgiz* or kirghiz* or laos or lao or laotian* or latvia* or lebanon* or lebanese* or lesotho* or lesothan* or lesothonian* or basutoland* or mosotho* or basotho* or liberia* or libya* or jamahiriya* or lithuania* or macedonia* or madagasca* or malagasy* or malawi* or nyasaland* or malaysia* or malay* federation or maldives* or maldivian* or indian ocean or mali or malian* or "mali's" or malta or maltese* or "malta's" or micronesia* or marshallese* or kiribati* or marshall island* or nauru or nauran or nauruans or "naurian's" or mariana or marianas or palau or paluan* or tuvalu* or mauritania* or mauritan* or mauritius* or mexico* or mexican* or moldova* or moldovia* or mongol* or montenegr* or morocco* or moroccan* or ifni or mozambique* or mozambican* or myanmar* or burma* or burmese or namibia* or nepal* or new caledonia* or netherlands antill* or nicaragua* or niger* or oman or omani or omanis or "oman's" or pakistan* or palestin* or gaza* or west bank* or panama* or paraguay* or peru or peruvian* or "peru's" or philippine* or philipine* or phillipine* or phillippine* or filipino* or filipina* or poland* or polish or pole or poles or portugal* or portuguese or puerto ric* or romania* or russia* or ussr* or soviet* or rwanda* or rwandese or ruanda* or ruandese or samoa* or navigator island* or pacific island* or polynesia* or sao tome* or santomean* or saudi arabia* or saudi or saudis or senegal* or serbia* or seychell* or sierra leone* or slovak* or sloven* or melanesia* or solomon island* or norfolk island* or somali* or sri lanka* or ceylon* or saint kitts or st kitts or kittian* or nevisian* or saint lucia* or st lucia* or saint vincent* or st vincent* or vincentian* or grenadine* or sudan* or surinam* or syria* or tajik* or tadjik* or tadzhik* or tanzania* or tanganyika* or thai* or timor leste* or east timor* or timorese* or togo or togoles* or "togo's" or tonga* or trinidad* or tobago* or tunisia* or turkiy* or turkey* or turk or turks or turkish or turkmen* or uganda* or ukrain* or uruguay* or uzbek* or vanuatu* or new hebrides* or venezuela* or vietnam* or viet nam* or yemen* or yugoslav* or zambia* or zimbabwe* or rhodesia* or arab* countr* or middle east* or global south or sahara* or subsahara* or magreb* or maghrib* or west indies* or caribbean* or central america* or latin america* or south america* or asia central or central asia* or asia northern or north asia* or northern asia* or asia southeastern or southeastern asia* or south eastern asia* or southeast asia* or south east asia* or asia western or west asia* or western asia* or europe eastern or east europe* or eastern europe* or developing countr* or developing nation* or developing population* or developing world or less developed countr* or less developed nation* or less developed world or lesser developed countr* or lesser developed nation* or lesser developed world or under developed countr* or under developed nation* or under developed world or underdeveloped countr* or underdeveloped nation* or underdeveloped world or middle income countr* or middle income nation* or middle income population* or low income countr* or low income nation* or low income population* or lower income countr* or lower income nation* or lower income population* or underserved countr* or underserved nation* or underserved population* or under served population* or under served nation* or under served population* or deprived countr* or deprived population* or high burden countr* or high burden nation* or countdown countr* or countdown nation* or poor countr* or poor nation* or poor population* or poor world or poorer countr* or poorer nation* or poorer population* or poorer world or developing econom* or less developed econom* or underdeveloped econom* or under developed econom* or middle income econom* or low income econom* or lower income econom* or low gdp or low gnp or low gross domestic or low gross national or lower gdp or lower gnp or lower gross domestic or lower gross national or lmic or lmics or third world or lami countr* or transitional countr* or emerging econom* or emerging nation*).ti,ab,hw,id. |

| Search Strategies—Web of Science and SCOPUS | | | |
| --- | --- | --- | --- |
| Database | Population/Exposure | Intervention | Setting |
| Web of Science | ("bipolar dis*" NEAR/2 psychos?s) OR (bipolar NEAR/2 psychos?s) OR ("bipolar depress*" NEAR/2 psychos?s) OR ("affective psychos?s") OR ("bipolar affective psychos?s") OR ("bipolar mood dis*" NEAR/2 psychos?s) OR ("bipolar affective dis*" NEAR/2 psychos?s) OR ("manic psychos?s") OR (mania NEAR/2 psychos?s) OR (BD NEAR/2 psychos?s) OR ("manic depress* psychos?s") OR ("bipolar dis*" NEAR/2 psychot*) OR (bipolar NEAR/2 psychot*) OR ("bipolar depress*" NEAR/2 psychot*) OR ("manic depress*" NEAR/2 psychot*) OR ("affective psychot*") OR ("affective disorder" NEAR/2 psychot*) OR ("bipolar mood disorder" NEAR/2 psychot*) OR ("bipolar affective dis*" NEAR/2 psychot*) OR (mania NEAR/2 psychot*) OR ("BPAD" NEAR/2 psychot*) OR ("BD" NEAR/2 psychot*) OR ("mood disorder" NEAR/2 psychot*) OR (depress* NEAR/2 psychos?s) OR ("major depress*" NEAR/2 psychos?s) OR ("MDD" NEAR/2 psychot*) OR (depress* NEAR/2 psychot*) OR ("major depress*" NEAR/2 psychot*) OR ("MDD" NEAR/2 psychot*) OR ("psychot* depress*") OR (schizophren*) OR (schizotyp*) OR ("schizo affect*") OR (schizoaffect*) OR (psychos?s) OR (psychot*) OR ("severe mental illnes*") OR ("severe mental dis*") OR ("serious mental illnes*") OR ("serious mental dis*") (Topic) | ("cognitive remediation" OR "cognitive rehab*" OR "cognitive training" OR "cognitive retraining" OR "brain training" OR "cognitive enhancement") (Topic) | (afghan* OR africa* OR albania* OR algeria* OR angola* OR antigua* OR barbuda* OR argentin* OR armenia* OR aruba* OR azerbaijan* OR bahrain* OR bangladesh* OR bengal* OR bangal* OR barbados* OR barbadian* OR bejan OR bejans OR belarus* OR belorus* OR byelarus* OR byelorus* OR belize* OR benin* OR dahomey OR bhutan* OR bolivia* OR bosnia* OR herzegovin* OR botswan* OR batswan* OR bechuanaland* OR brazil* OR brasil* OR bulgaria* OR burkina* OR burkinese* OR "upper volta*" OR burundi* OR urundi* OR "cabo verde*" OR "cape verde*" OR cambodia* OR kampuchea* OR khmer* OR cameroon* OR cameroun* OR "ubangi shari*" OR chad* OR chile* OR china* OR chinese OR colombia* OR comoro* OR comore* OR comorian* OR mayotte* OR congo* OR zaire* OR "costa rica*" OR "cote d'ivoir*" OR "cote d' ivoir*" OR "cote divoir*" OR "cote d ivoir*" OR "ivory coast*" OR ivorian* OR croatia* OR cuba OR cuban OR cubans OR "cuba's" OR cyprus* OR cypriot* OR czech* OR djibouti* OR "french somaliland*" OR dominica* OR ecuador* OR egypt* OR "united arab republic*" OR "el salvador*" OR salvadoran* OR guinea* OR equatoguinea* OR eritrea* OR estonia* OR eswatini* OR swaziland* OR swazi* OR swati* OR ethiopia* OR fiji* OR gabon* OR gabonese* OR gabonaise* OR gambia* OR ((georgia OR georgian OR georgians) NOT (atlanta OR california OR florida)) OR ghana* OR gibraltar* OR greece* OR greek* OR grecian* OR grenada* OR grenadian* OR guam* OR guatemala* OR guyana* OR guiana* OR guyanese* OR haiti* OR hispaniola* OR hondura* OR hungary* OR hungarian* OR india* OR indonesia* OR iran* OR iraq* OR "isle of man*" OR jamaica* OR jordan* OR kazakh* OR kenya* OR karabati* OR korea* OR kosovo* OR kosova* OR kyrgyz* OR kirgiz* OR kirghiz* OR laos OR lao OR laotian* OR latvia* OR lebanon* OR lebanese* OR lesotho* OR lesothan* OR lesothonian* OR basutoland* OR mosotho* OR basotho* OR liberia* OR libya* OR jamahiriya* OR lithuania* OR macedonia* OR madagasca* OR malagasy* OR malawi* OR nyasaland* OR malaysia* OR "malay* federation" OR maldives* OR maldivian* OR "indian ocean" OR mali OR malian* OR "mali's" OR malta OR maltese* OR "malta's" OR micronesia* OR marshallese* OR kiribati* OR "marshall island*" OR nauru OR najran OR nauruan OR "naurian's" OR mariana OR marianas OR palau OR paluan* OR tuvalu* OR mauritania* OR mauritan* OR mauritius* OR mexico* OR mexican* OR moldova* OR moldovia* OR mongol* OR montenegr* OR morocco* OR moroccan* OR ifni OR mozambique* OR mozambican* OR myanmar* OR burma* OR burmese OR namibia* OR nepal* OR "new caledonia*" OR "netherlands antill*" OR nicaragua* OR niger* OR oman OR omani OR omanis OR "oman's" OR pakistan* OR palestin* OR gaza* OR "west bank*" OR panama* OR paraguay* OR peru OR peruvian* OR "peru's" OR philippine* OR philipine* OR phillipine* OR phillippine* OR filipino* OR filipina* OR poland* OR polish OR pole OR poles OR portugal* OR portuguese OR "puerto ric*" OR romania* OR russia* OR ussr* OR soviet* OR rwanda* OR randes OR ruanda* OR rwandese OR samoa* OR "navigator island*" OR "pacific island*" OR polynesia* OR "sao tome*" OR santomean* OR "saudi arabia*" OR saudi OR saudis OR senegal* OR serbia* OR seychell* OR "sierra leone*" OR slovak* OR sloven* OR melanesia* OR "solomon island*" OR "norfolk island*" OR somali* OR "sri lanka*" OR ceylon* OR "saint kitts" OR "st kitts" OR kittian* OR nevisian* OR "saint lucia*" OR "st lucia*" OR "saint vincent*" OR "st vincent*" OR vincentian* OR grenadine* OR sudan* OR surinam* OR syria* OR tajik* OR tadjik* OR tadzhik* OR tanzania* OR tanganyika* OR thai* OR "timor leste*" OR "east timor*" OR timorese* OR togo OR togoles* OR "togo's" OR tonga* OR trinidad* OR tobago* OR tunisia* OR turkiy* OR turkey* OR turk OR turks OR turkish OR turkmen* OR uganda* OR ukrain* OR uruguay* OR uzbek* OR vanuatu* OR "new hebrides*" OR venezuela* OR vietnam* OR "viet nam*" OR yemen* OR yugoslav* OR zambia* OR zimbabwe* OR rhodesia* OR "arab* countr*" OR "middle east*" OR "global south" OR sahara* OR subsahara* OR magreb* OR maghrib* OR "west indies*" OR caribbean* OR "central america*" OR "latin america*" OR "south america*" OR "asia central" OR "central asia*" OR "asia northern" OR "north asia*" OR "northern asia*" OR "asia southeastern" OR "southeastern asia*" OR "south eastern asia*" OR "southeast asia*" OR "south east asia*" OR "asia western" OR "west asia*" OR "western asia*" OR "europe eastern" OR "east europe*" OR "eastern europe*" OR "developing countr*" OR "developing nation*" OR "developing population*" OR "developing world" OR "less developed countr*" OR "less developed nation*" OR "less developed world" OR "lesser developed countr*" OR "lesser developed nation*" OR "lesser developed world" OR "under developed countr*" OR "under developed nation*" OR "under developed world" OR "underdeveloped countr*" OR "underdeveloped nation*" OR "underdeveloped world" OR "middle income countr*" OR "middle income nation*" OR "middle income population*" OR "low income countr*" OR "low income nation*" OR "low income population*" OR "lower income countr*" OR "lower income nation*" OR "lower income population*" OR "underserved countr*" OR "underserved nation*" OR "underserved population*" OR "under served population*" OR "under served nation*" OR "under served population*" OR "deprived countr*" OR "deprived population*" OR "high burden countr*" OR "high burden nation*" OR "countdown countr*" OR "countdown nation*" OR "poor countr*" OR "poor nation*" OR "poor population*" OR "poor world" OR "poorer countr*" OR "poorer nation*" OR "poorer population*" OR "poorer world" OR "developing econom*" OR "less developed econom*" OR "underdeveloped econom*" OR "under developed econom*" OR "middle income econom*" OR "low income econom*" OR "lower income econom*" OR "low gdp" OR "low gnp" OR "low gross domestic" OR "low gross national" OR "lower gdp" OR "lower gnp" OR "lower gross domestic" OR "lower gross national" OR lmic OR lmics OR "third world" OR "lami countr*" OR "transitional countr*" OR "emerging econom*" OR "emerging nation*") (Topic) |
| SCOPUS | ( "bipolar dis*" W/2 psychos?s ) OR ( bipolar W/2 psychos?s ) OR ( "bipolar depress*" W/2 psychos?s ) OR ( "affective psychos?s" ) OR ( "bipolar affective psychos?s" ) OR ( "bipolar mood dis*" W/2 psychos?s ) OR ( "bipolar affective dis*" W/2 psychos?s ) OR ( "manic psychos?s" ) OR ( mania W/2 psychos?s ) OR ( bd W/2 psychos?s ) OR ( "manic depress* psychos?s" ) OR ( "bipolar dis*" W/2 psychot* ) OR ( bipolar W/2 psychot* ) OR ( "bipolar depress*" W/2 psychot* ) OR ( "manic depress*" W/2 psychot* ) OR ( "affective psychot*" ) OR ( "affective disorder" W/2 psychot* ) OR ( "bipolar mood disorder" W/2 psychot* ) OR ( "bipolar affective dis*" W/2 psychot* ) OR ( mania W/2 psychot* ) OR ( "BPAD" W/2 psychot* ) OR ( "BD" W/2 psychot* ) OR ( "mood disorder" W/2 psychot* ) OR ( depress* W/2 psychos?s ) OR ( "major depress*" W/2 psychos?s ) OR ( "MDD" W/2 psychot* ) OR ( depress* W/2 psychot* ) OR ( "major depress*" W/2 psychot* ) OR ( "psychot* depress*" ) OR ( schizophren* ) OR ( schizotyp* ) OR ( "schizo affect*" ) OR ( psychos?s ) OR ( psychot* ) OR ( "severe mental illnes*" ) OR ( "severe mental dis*" ) OR ( "serious mental illnes*" ) OR ( "serious mental dis*" ) | ( "cognitive remediation" ) OR ( "cognitive rehabilitation" ) OR ( "cognitive training" ) OR ( "cognitive retraining" ) OR ( "brain training" ) OR ( "cognitive enhancement" ) | ( afghan* OR africa* OR albania* OR algeria* OR angola* OR antigua* OR barbuda* OR argentin* OR armenia* OR aruba* OR azerbaijan* OR bahrain* OR bangladesh* OR bengal* OR bangal* OR barbados* OR barbadian* OR bajan OR bajans OR belarus* OR belorus* OR byelarus* OR byelorus* OR belize* OR benin* OR dahomey OR bhutan* OR bolivia* OR bosnia* OR herzegovin* OR botswan* OR batswan* OR bechuanaland* OR brazil* OR brasil* OR bulgaria* OR burkina* OR burkinese* OR "upper volta*" OR burundi* OR urundi* OR "cabo verde*" OR "cape verde*" OR cambodia* OR kampuchea* OR khmer* OR cameroon* OR cameroun* OR "ubangi shari*" OR chad* OR chile* OR china* OR chinese OR colombia* OR comoro* OR comore* OR comorian* OR mayotte* OR congo* OR zaire* OR "costa rica*" OR "cote d&apos;ivoir*" OR "cote d&apos; ivoir*" OR "cote divoir*" OR "cote d ivoir*" OR "ivory coast*" OR ivorian* OR croatia* OR cuba OR cuban OR cubans OR "cuba&apos;s" OR cyprus* OR cypriot* OR czech* OR djibouti* OR "french somaliland*" OR dominica* OR ecuador* OR egypt* OR "united arab republic*" OR "el salvador*" OR salvadoran* OR guinea* OR equatoguinea* OR eritrea* OR estonia* OR eswatini* OR swaziland* OR swazi* OR swati* OR ethiopia* OR fiji* OR gabon* OR gabonese* OR gabonaise* OR gambia* OR ( ( georgia OR georgian OR georgians ) AND NOT ( atlanta OR california OR florida ) ) OR ghana* OR gibraltar* OR greece* OR greek* OR grecian* OR grenada* OR grenadian* OR guam* OR guatemala* OR guyana* OR guiana* OR guyanese* OR haiti* OR hispaniola* OR hondura* OR hungary* OR hungarian* OR india* OR indonesia* OR iran* OR iraq* OR "isle of man*" OR jamaica* OR jordan* OR kazakh* OR kenya* OR karabati* OR korea* OR kosovo* OR kosova* OR kyrgyz* OR kirgiz* OR kirghiz* OR laos OR lao OR laotian* OR latvia* OR lebanon* OR lebanese* OR lesotho* OR lesothan* OR lesothonian* OR basutoland* OR mosotho* OR basotho* OR liberia* OR libya* OR jamahiriya* OR lithuania* OR macedonia* OR madagasca* OR malagasy* OR malawi* OR nyasaland* OR malaysia* OR "malay* federation" OR maldives* OR maldivian* OR "indian ocean" OR mali OR malian* OR "mali&apos;s" OR malta OR maltese* OR "malta&apos;s" OR micronesia* OR marshallese* OR kiribati* OR "marshall island*" OR nauru OR nauran OR nauruans OR "naurian&apos;s" OR mariana OR marianas OR palau OR paluan* OR tuvalu* OR mauritania* OR mauritan* OR mauritius* OR mexico* OR mexican* OR moldova* OR moldovia* OR mongol* OR montenegr* OR morocco* OR moroccan* OR ifni OR mozambique* OR mozambican* OR myanmar* OR burma* OR burmese OR namibia* OR nepal* OR "new caledonia*" OR "netherlands antill*" OR nicaragua* OR niger* OR oman OR omani OR omanis OR "oman&apos;s" OR pakistan* OR palestin* OR gaza* OR "west bank*" OR panama* OR paraguay* OR peru OR peruvian* OR "peru&apos;s" OR philippine* OR philipine* OR phillipine* OR phillippine* OR filipino* OR filipina* OR poland* OR polish OR pole OR poles OR portugal* OR portuguese OR "puerto ric*" OR romania* OR russia* OR ussr* OR soviet* OR rwanda* OR rwandese OR ruanda* OR ruandese OR samoa* OR "navigator island*" OR "pacific island*" OR polynesia* OR "sao tome*" OR santomean* OR "saudi arabia*" OR saudi OR saudis OR senegal* OR serbia* OR seychell* OR "sierra leone*" OR slovak* OR sloven* OR melanesia* OR "solomon island*" OR "norfolk island*" OR somali* OR "sri lanka*" OR ceylon* OR "saint kitts" OR "st kitts" OR kittian* OR nevisian* OR "saint lucia*" OR "st lucia*" OR "saint vincent*" OR "st vincent*" OR vincentian* OR grenadine* OR sudan* OR surinam* OR syria* OR tajik* OR tadjik* OR tadzhik* OR tanzania* OR tanganyika* OR thai* OR "timor leste*" OR "east timor*" OR timorese* OR togo OR togoles* OR "togo&apos;s" OR tonga* OR trinidad* OR tobago* OR tunisia* OR turkiy* OR turkey* OR turk OR turks OR turkish OR turkmen* OR uganda* OR ukrain* OR uruguay* OR uzbek* OR vanuatu* OR "new hebrides*" OR venezuela* OR vietnam* OR "viet nam*" OR yemen* OR yugoslav* OR zambia* OR zimbabwe* OR rhodesia* OR "arab* countr*" OR "middle east*" OR "global south" OR sahara* OR subsahara* OR magreb* OR maghrib* OR "west indies*" OR caribbean* OR "central america*" OR "latin america*" OR "south america*" OR "asia central" OR "central asia*" OR "asia northern" OR "north asia*" OR "northern asia*" OR "asia southeastern" OR "southeastern asia*" OR "south eastern asia*" OR "southeast asia*" OR "south east asia*" OR "asia western" OR "west asia*" OR "western asia*" OR "europe eastern" OR "east europe*" OR "eastern europe*" OR "developing countr*" OR "developing nation*" OR "developing population*" OR "developing world" OR "less developed countr*" OR "less developed nation*" OR "less developed world" OR "lesser developed countr*" OR "lesser developed nation*" OR "lesser developed world" OR "under developed countr*" OR "under developed nation*" OR "under developed world" OR "underdeveloped countr*" OR "underdeveloped nation*" OR "underdeveloped world" OR "middle income countr*" OR "middle income nation*" OR "middle income population*" OR "low income countr*" OR "low income nation*" OR "low income population*" OR "lower income countr*" OR "lower income nation*" OR "lower income population*" OR "underserved countr*" OR "underserved nation*" OR "underserved population*" OR "under served population*" OR "under served nation*" OR "under served population*" OR "deprived countr*" OR "deprived population*" OR "high burden countr*" OR "high burden nation*" OR "countdown countr*" OR "countdown nation*" OR "poor countr*" OR "poor nation*" OR "poor population*" OR "poor world" OR "poorer countr*" OR "poorer nation*" OR "poorer population*" OR "poorer world" OR "developing econom*" OR "less developed econom*" OR "underdeveloped econom*" OR "under developed econom*" OR "middle income econom*" OR "low income econom*" OR "lower income econom*" OR "low gdp" OR "low gnp" OR "low gross domestic" OR "low gross national" OR "lower gdp" OR "lower gnp" OR "lower gross domestic" OR "lower gross national" OR lmic OR lmics OR "third world" OR "lami countr*" OR "transitional countr*" OR "emerging econom*" OR "emerging nation*" ) |
