# Supplementary material for: Cognitive remediation therapy in low-and-middle-income countries (LAMICs) for people with psychosis: a systematic review of its efficacy on functional and cognitive outcomes
Source: Soc Psychiatry Psychiatr Epidemiol. 2026 Jun 11;61(8):1361–86. doi: 10.1007/s00127-026-03136-x (PMC13522050; doi:10.1007/s00127-026-03136-x)
Supplement: Supplementary file 3 [file 127_2026_3136_MOESM3_ESM.docx]

Online Resource 3: Inclusion and Exclusion Criteria

*Social Psychiatry and Psychiatric Epidemiology*

**Cognitive Remediation Therapy in Low-and-Middle-Income Countries (LAMICs): A systematic review of its efficacy on functional and cognitive outcomes**

Yamini, Vig ^1,2,3^; Yohannes Gebreegziabhere 4,5; Charlotte, Hanlon 4,6; Matteo, Cella1,7.

1 - Institute of Psychiatry, Psychology and Neuroscience, King's College London, London, UK.

2 - Centre for Global Mental Health, London School of Hygiene and Tropical Medicine, London, UK.

3 - Addictions and Related Research Group (ARG), Sangath, Porvorim, Goa, India. Email: [yaminivig16@gmail.com](mailto:yaminivig16@gmail.com). ORCID: 0009-0001-9288-5939

4 - Department of Psychiatry, College of Health Sciences, Addis Ababa University, Addis Ababa, Ethiopia.

5 - Department of Nursing, College of Health Sciences, Debre Berhan University, Debre Berhan, Ethiopia. Email: [yohannes36@gmail.com](mailto:yohannes36@gmail.com).

6 - Division of Psychiatry, Centre for Clinical Brain Sciences. The University of Edinburgh, Edinburgh, Scotland, UK; and Department of Psychiatry, School of Medicine, College of Health Sciences, Addis Ababa University, Addis Ababa, Ethiopia. Email: [chanlon@ed.ac.uk](mailto:chanlon@ed.ac.uk). ORCID: 0000-0002-7937-3226

7 - South London and Maudsley NHS Foundation Trust, London, UK. 0000-0002-5701-0336

| Table S2. Inclusion and Exclusion Criteria | |
| --- | --- |
| **Population** | |
| **Inclusion:**   i. Adults aged ≥ 18 years. ii. Evidence of severe mental illness based on standardised diagnostic instruments or criteria, or via clinical assessments. iii. Severe mental illness operationalised as schizophrenia, schizoaffective disorder, schizophreniform disorder, delusional disorder, mood disorders with psychotic features (bipolar affective disorders or depression), postpartum psychosis or any other psychotic disorder associated with disability. iv. People with comorbid disorders (e.g., anxiety and substance use) in addition to SMI were included. v. Participants were residents of low-and middle-income countries (based on the World Bank classification of the country at the time of study publication. | **Exclusion:**   i. Studies with participants experiencing prodromal psychosis, subclinical symptoms, or at risk/ultra-high risk of experiencing psychosis, i.e., a clear diagnosis of psychosis (schizophrenia/schizoaffective disorder) was not present. ii. People suffering from dementia, traumatic brain injuries, and other conditions impacting cognition. |
| **Intervention** | |
| **Inclusion:**  Cognitive remediation has been defined as “an intervention targeting cognition* using scientific principles of learning with the goal of improving functional outcomes. Its effectiveness is enhanced when provided in a context (formal or informal) that provides support and opportunity for extending everyday functioning.” [Cognitive Remediation Experts Workshop 2023]  *Attention, memory, executive function, social cognition, or metacognition  This included:   i. Interventions based on the principles of cognitive remediation therapy (CRT), targeting cognitive and functional outcomes ii. Either Pen-paper, i.e., manualised CRT or computerised/software-delivered interventions iii. Delivered by trained professionals, trained caregivers, and people trained to administer CRT tasks or software delivered. iv. The interventions could be focused on drill and practice, and/or emphasise the use of cognitive strategies. v. Including the following active ingredients: errorless learning, massed practice, problem-solving strategies, contact and support from a therapist. vi. Target cognitive areas including but not limited to attention, memory, executive function, metacognition, etc. | **Exclusion:**  The review did not include   i. mindfulness-based interventions aiming to improve cognitive functioning as the primary   intervention,   ii. Cognitive Behaviour Therapy as the primary intervention or iii. Any experimental/lab-based studies that did not aim to translate the intervention for use in routine healthcare settings. |
| **Control** | |
| **Inclusion:**  Control conditions could include treatment as usual, enhanced treatment as usual, passive/active control, waitlists, and pre-post assessments. | **Exclusion:**  Studies with variations of cognitive remediation therapy as the control condition were excluded. |
| **Outcomes** | |
| **Inclusion:**  **Primary outcomes:**   i. Cognitive outcomes:    - Cognitive outcomes could be based on global cognition or specific areas of cognition, i.e., memory (working, verbal, non-verbal, spatial, etc.), attention, executive functioning, and metacognition. They needed to have been measured using standardised assessment tools/tests. For example, (not limited to) the Rey Osterrieth Complex Figure, Trail Making Test, etc. Studies could include multiple tasks to assess various cognitive domains or cognitive assessment interviews.   ii Functional outcomes   - Functional outcomes could be self-reported, caregiver-reported, or based on a clinician’s report - Functional outcomes needed to be measured using standardised assessment tools. E.g., WHODAS 2.0. - Functional outcomes could encompass the domains of independent living (e.g., activities of daily living), economic (education/career related and occupational achievement) or social functioning.   **Secondary Outcomes:**   i. Adverse Events and/or drop-outs/attrition:   Any adverse events reported throughout the intervention period and follow-up duration as well as dropouts throughout the study duration.   ii. Quality of life   Measures of effect  Measures of effect (intervention vs. control or pre-post) could include risk ratios, odds ratios, mean difference, beta coefficient or Cohen’s d (standardised mean difference).  Using standardised assessment tools  Additional outcome(s)  Percentage experiencing adverse events and/or drop-outs in intervention vs. control arms  Measures of effect  Drop-out rates, proportion of adverse events and standardised measures for Quality of Life. | |
| **Study Design** | |
| **Inclusion:**  Intervention/experimental study designs:   i. Randomised control trials ii. Non-randomised controlled trials iii. Pre-post studies iv. Quasi-experimental studies v. Interrupted time series vi. Pilot intervention studies | **Exclusion:**   i. Observational quantitative studies (cross-sectional, case-control, control), qualitative studies, psychometric assessments/studies, conference abstracts or posters, theses, commentaries, editorials, and study proposals/protocols. ii. Economic evaluation studies, reviews or commentaries. |
| **Setting** | |
| **Inclusion:**  Any context based within an LAMIC (as defined by the World Bank Classification). The platform of care could include hospitals, primary care or specialist settings, community-based settings, educational settings, and/or religious/spiritual places of healing (primary, secondary, tertiary, and informal places of care). | **Exclusion:**  Lab-based without the intention to generalise and use findings for wider healthcare |
| CRT, Cognitive Remediation Therapy; WHODAS 2.0, WHO Disability Assessment Schedule 2.0; LAMIC, Low and Middle-Income Countries | |
