# Supplementary material for: Cognitive remediation therapy in low-and-middle-income countries (LAMICs) for people with psychosis: a systematic review of its efficacy on functional and cognitive outcomes
Source: Soc Psychiatry Psychiatr Epidemiol. 2026 Jun 11;61(8):1361–86. doi: 10.1007/s00127-026-03136-x (PMC13522050; doi:10.1007/s00127-026-03136-x)
Supplement: Supplementary file 4 [file 127_2026_3136_MOESM5_ESM.docx]

Online Resource 5: Traffic Light Plots (Fig. S1 Risk of Bias Assessment Summary using ROB 2.0 and Fig. S2 Risk of Bias Assessment Summary using ROBINS-I)

*Social Psychiatry and Psychiatric Epidemiology*

**Cognitive Remediation Therapy in Low-and-Middle-Income Countries (LAMICs): A systematic review of its efficacy on functional and cognitive outcomes**

Yamini, Vig ^1,2,3^; Yohannes Gebreegziabhere 4,5; Charlotte, Hanlon 4,6; Matteo, Cella1,7.

1 - Institute of Psychiatry, Psychology and Neuroscience, King's College London, London, UK.

2 - Centre for Global Mental Health, London School of Hygiene and Tropical Medicine, London, UK.

3 - Addictions and Related Research Group (ARG), Sangath, Porvorim, Goa, India. Email: [yaminivig16@gmail.com](mailto:yaminivig16@gmail.com). ORCID: 0009-0001-9288-5939

4 - Department of Psychiatry, College of Health Sciences, Addis Ababa University, Addis Ababa, Ethiopia.

5 - Department of Nursing, College of Health Sciences, Debre Berhan University, Debre Berhan, Ethiopia. Email: [yohannes36@gmail.com](mailto:yohannes36@gmail.com).

6 - Division of Psychiatry, Centre for Clinical Brain Sciences. The University of Edinburgh, Edinburgh, Scotland, UK; and Department of Psychiatry, School of Medicine, College of Health Sciences, Addis Ababa University, Addis Ababa, Ethiopia. Email: [chanlon@ed.ac.uk](mailto:chanlon@ed.ac.uk). ORCID: 0000-0002-7937-3226

7 - South London and Maudsley NHS Foundation Trust, London, UK. 0000-0002-5701-0336

**Fig. S1** Risk of Bias Assessment Summary using ROB 2.0

**Fig. S2** Risk of Bias Assessment Summary using ROBINS-I
